# Current practice on covariate adjustment and stratified analysis —based on survey results by ASA oncology estimand working group conditional and marginal effect task force

**DOI:** 10.1186/s12874-025-02670-7

**Published:** 2025-11-04

**Authors:** Jiawei Wei, Sarwar I. Mozumder, Liming Li, Dong Xi, Jiajun Xu, Ray Lin, Oleksandr Sverdlov, Jonathan J. Chipman, Conditional, Marginal Task Force under ASA Oncology Estimand Working Group

**Affiliations:** 1 Novartis Institutes for Biomedical Research Co., Shanghai, China; 2 Statistics and Data Science Innovation Hub, GSK Research and Development, London, UK; 3 AstraZeneca, Shanghai, China; 4 Gilead Sciences, Foster City, USA; 5 Johnson & Johnson Innovative Medicine (China), Shanghai, China; 6 Genentech/Roche, South San Francisco, USA; 7 Novartis Pharmaceuticals Corporation, East Hanover, USA; 8 Division of Biostatistics, University of Utah Intermountain Healthcare Department of Population Health Sciences, University of Utah, Salt Lake City, USA

**Keywords:** Covariate adjustment, Stratified analysis, Estimand, Survey

## Abstract

**Background:**

The 2023 FDA's guidance on covariate adjustment encourages the judicious use of baseline covariates to enhance efficiency. However, when performing covariate adjustment in non-linear models, care must be taken on preserving estimation of the target estimand as introduced by the ICH E9(R1) addendum. To understand the current practices of covariate adjustment within the context of the estimands framework across various sectors and associated challenges, the conditional and marginal effect task force within the ASA Oncology Estimand working group conducted a survey.

**Methods:**

The target participants of the survey were biostatisticians who support study designs and analyses in clinical trials in the drug development industry or in academia. A total of 19 questions were included in an online survey that was distributed between June and July 2023. The survey was disseminated via a shared online link to contacts from more than 50 organisations. The survey response and experience from the working group on challenges of covariate adjustment and stratified analysis are summarized and discussed in detail.

**Results:**

A total of 122 responses were received from 12 countries. The survey results suggest that there remain gaps in the understanding of different statistical analysis models which may target different estimands for non-collapsable measures, highlighting the need for further clarification and training on this topic. In terms of general practice, when performing the analysis under stratified randomization, additional covariates may be added in the analysis model beyond those used for stratifying randomization, and small strata may be pooled to avoid the estimation challenges.

**Conclusions:**

This paper summarises the results from this survey and based on our findings, we provide some recommendations to establish consistency and clarifications on any widely misunderstood practices.

**Supplementary Information:**

The online version contains supplementary material available at 10.1186/s12874-025-02670-7.

## Background

Since the addendum to the ICH E9 guidelines, there has been a shift towards explicitly defining the clinical question and treatment effect of interest [1]. This requires pre-specification of the target estimand, independent of the estimation approach, as highlighted in the additional FDA guidelines released in May 2023 on “Adjusting for Covariates in Randomized Clinical Trials for Drugs and Biological Products” [1]. Covariate adjustment impacts the population summary measure attribute of the estimand and the subsequent interpretation of the treatment effect, according to Morris et al. [2]. The chosen summary measure must be correctly estimated within the respective analysis method of the estimator to obtain the target estimand that addresses the intended clinical question. The choice between conditional and marginal estimands depends on whether the researcher is interested in individual treatment effects with specific characteristics or the average treatment effect in a population, as discussed in Morris et al. [2] and Daniel et al. [4].

Once the estimand and clinical question of interest are identified, the estimator and the analysis method that estimates the target estimand must be defined. For instance, in time-to-event endpoint trials, a stratified Cox regression model is typically specified within studies utilizing stratified randomization, where stratified hazard ratios are chosen as the population summary measure to account for each specified stratum. Previously, the estimator and analysis method were commonly defined without making the target estimand clear, but it is now crucial to understand and identify whether it is the marginal or conditional estimand that is of primary interest. How this is targeted may then be followed by the appropriate analysis method, which may be via a stratified or an unstratified analysis. A stratified analysis always targets the conditional estimand, while an unstratified analysis may target a conditional or a marginal estimand depending on the question of interest.

With the release of the ICH E9(R1) [8] and the FDA guidance on covariate adjustment, there is a need to evaluate the current landscape and understanding of covariate adjustment within the context of estimands and how this is targeted in estimation, to identify current gaps in guidance and implementation in practice. To address this, the conditional versus marginal effects task force within the American Statistical Association Oncology Estimand Working Group conducted a survey to identify gaps and areas that required clarification to address any misunderstanding or misinterpretation.

## Methods

### Survey design and distribution

The survey was set up on SurveyPlanet.com and was active between June and July 2023 for participants to complete. The target audience of this survey were biostatisticians who perform analyses and design studies within drug development/clinical trials. This included those in industry and in academia.

The survey was distributed amongst the Oncology Estimand WG members and point-of-contacts at various companies and institutions and was also posted in the ASA Biopharm section. Optional anonymous submission of the survey was allowed to maximize participation with accurate responses.

### Types of questions

In this survey, we included 19 questions, which can be classified into five broad categories. Q1 to Q4 captures the characteristics of the survey participants. Q5, Q6 and Q15 are designed to understand how individuals define the estimand when specifying covariate-adjusted or stratified analyses. Q7, Q8 and Q9 focus on the estimator and analysis method, specifically, around the selection of stratification factors/covariates. Q10 to Q14 are designed to understand the challenges of small-strata issue. Finally, Q16 to Q19 collect the challenges associated with implementing covariate adjustment or stratification and any gaps that still need addressing. The survey with the full list of questions is provided in the Supplementary Material A along with the raw output of survey results from surveryplanet.com (Supplementary Material C).

## Results

### Characteristics of the respondents (Q1, Q2)

A total number of 122 respondents participated in the survey. Among those 122 respondents, 100(81.3%) work in confirmatory clinical trials, and 96(78.7%) come from pharmaceutical or biotech companies, 11(9.0%) from contract or consulting companies, 10(8.2%) from academic centers and 3(2.5%) from regulatory authorities. The countries represented in this survey span across the world, with nearly half (46.7%) of the respondents from the United States, followed by China (19.7%) and Switzerland (11.5%). Please refer to Supplementary Material B, Table S1 for more details.

### Availability of internal guidelines within the organization (Q3, Q4)

Among all these 122 respondents, the majority (59.8%) do not have or are not aware of a company-wide or disease-specific guidance on covariate adjustment or stratified analyses. Among those who have internal guidance (n = 49), over half of the respondents received guidance on both covariate adjustment and stratified analysis. Institutions providing guidance covers multiple therapeutic areas; however, most respondents (73.5%) reported having internal guidance within oncology. Please refer to Supplementary Material B, Table S2 and Figure S1 for more details.

### Does covariate adjustment or stratified analysis in a non-linear model target different estimands? (Q5, Q6, Q15)

As a result of research and various discussions of covariate adjustment and stratified analysis within the Oncology Estimand Working Group, it was established that there was some confusion on whether the stratified Cox model was a sensitivity analysis or a supplementary analysis to the unstratified (and unadjusted) Cox model [3]. This motivated for the three questions (Q5, Q6, Q15) in the survey to further explore the respondents’ current understanding on whether covariate adjustment or stratified analysis targets different estimands in the context of a non-linear model. Among the three questions, Q5 and Q6 are more general, where participants are asked whether stratified and unstratified analysis, or covariate-adjusted analysis and covariate-unadjusted analysis are targeting different estimands in non-linear models (such as Cox regression and logistic regression model). Q15 is concerned with a more practical setting to probe whether removing or pooling strata on an ad-hoc basis for interim analysis changes the pre-specified estimand for the final analysis.

Although this has been thoroughly discussed in the FDA guidelines [1] and elsewhere in literature for non-collapsible measures (for example, [4]), the survey result suggests that many respondents were still not aware that covariate adjusted or stratified analyses may estimate different quantities in a non-linear model. The results are summarized in Table 1. Among the 122 respondents, 61.5% respondents think that a stratified analysis and unstratified analysis target the same estimand in non-linear models; if one is chosen as the primary analysis, the other could be a "sensitivity analysis”, (which, by definition, targets the same estimand as the primary analysis). Similarly, 56.6% of respondents indicated that covariate-adjusted analysis and covariate-unadjusted analysis target the same estimand in non-linear models, and 57.4% respondents believe removing or pooling strata on an ad-hoc basis for interim analysis does not change the pre-specified estimand. The result is quite consistent across the three questions, which demonstrates the gap in the understanding of different statistical analysis models targeting different estimands for non-collapsible measures, and the critical need of further clarification and training on this topic. Retrospectively, we acknowledge that the survey question in relation to unstratified analyses may have been open to interpretation, since this can refer to an unadjusted analysis (which would always target a marginal estimand, or an adjusted analysis (which may target a conditional or marginal estimand in non-linear models depending on how it is approached in estimation). For clarity, our recommendations and conclusions insinuate that the unstratified analyses referred to unadjusted models. However, we highlight this as a limitation of the responses to the question in reference to unstratified analyses.

**Table 1:** Does covariate adjustment or stratified analysis in a non-linear model target different estimands? Summary of results from Q5, Q6 and Q15

Different non-linear models	Do they target the same estimand?	
	Yes	No
Stratified analysis vs. unstratified analysis	61.48% (75/122)	31.97% (39/122)
covariate-adjusted analysis vs. covariate-unadjusted analysis	56.56% (69/122)	38.52% (47/122)
Remove or pool strata at interim vs. prespecified analysis at final	57.38% (70/122)	38.52% (47/122)

These three questions are not mandatory, for each question there are non-responders

### How to select/include covariates or stratification factors in the model (Q7, Q8, Q9)

Both covariate adjustment and stratification are commonly used in clinical trials. Hence, we designed three questions (Q7-Q9) to understand the current practice of how to adjust for covariates and stratification factors when stratified randomization is used, and how to select covariates and stratification factors. As recommended by the FDA guideline [1], strata variables should be generally included in the covariate adjustment model. Additional covariates not used for stratifying randomization could also be included.

**Fig. 1:**
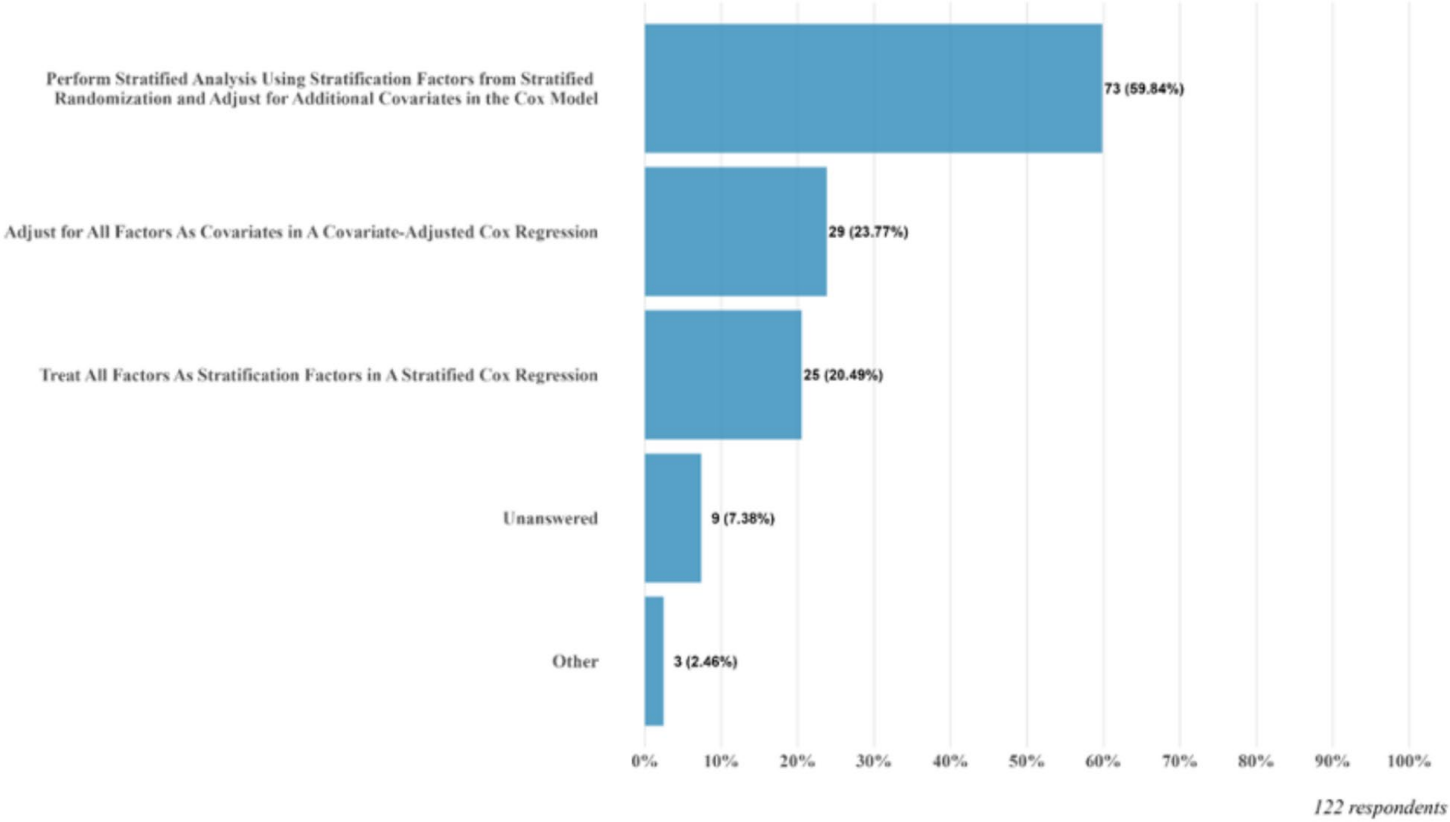
Response to Q8: In a trial with stratified randomization, when using Cox regression, how do you incorporate stratification factors from stratified randomization as well as other prognostic covariates in the model

Among these three questions, Q7 focuses on which variables should be included in a general analysis model in the context of stratified randomization and Q8 focuses on the specific analysis model of the Cox regression model. Among the respondents to Q7, 65.6% of them also consider adding additional covariates to be adjusted in the analysis model beyond those used for stratifying randomization, while 30.3% of them only consider factors used for stratified randomization in the analysis model. There are also 4.1% respondents without providing an answer to Q7. We observed a similar pattern from responses of Q8. The results are summarized in Fig. 1. There are 59.8% of respondents who consider including stratification factors in the stratified analysis and adjusting for additional covariates in the Cox model. There are 28.7% respondents who would include stratification factors in the stratified analysis and 23.8% respondents who would adjust for all factors as covariates. Thus, most of the respondents are following the recommendations from the FDA guidance [1].

**Fig. 2:**
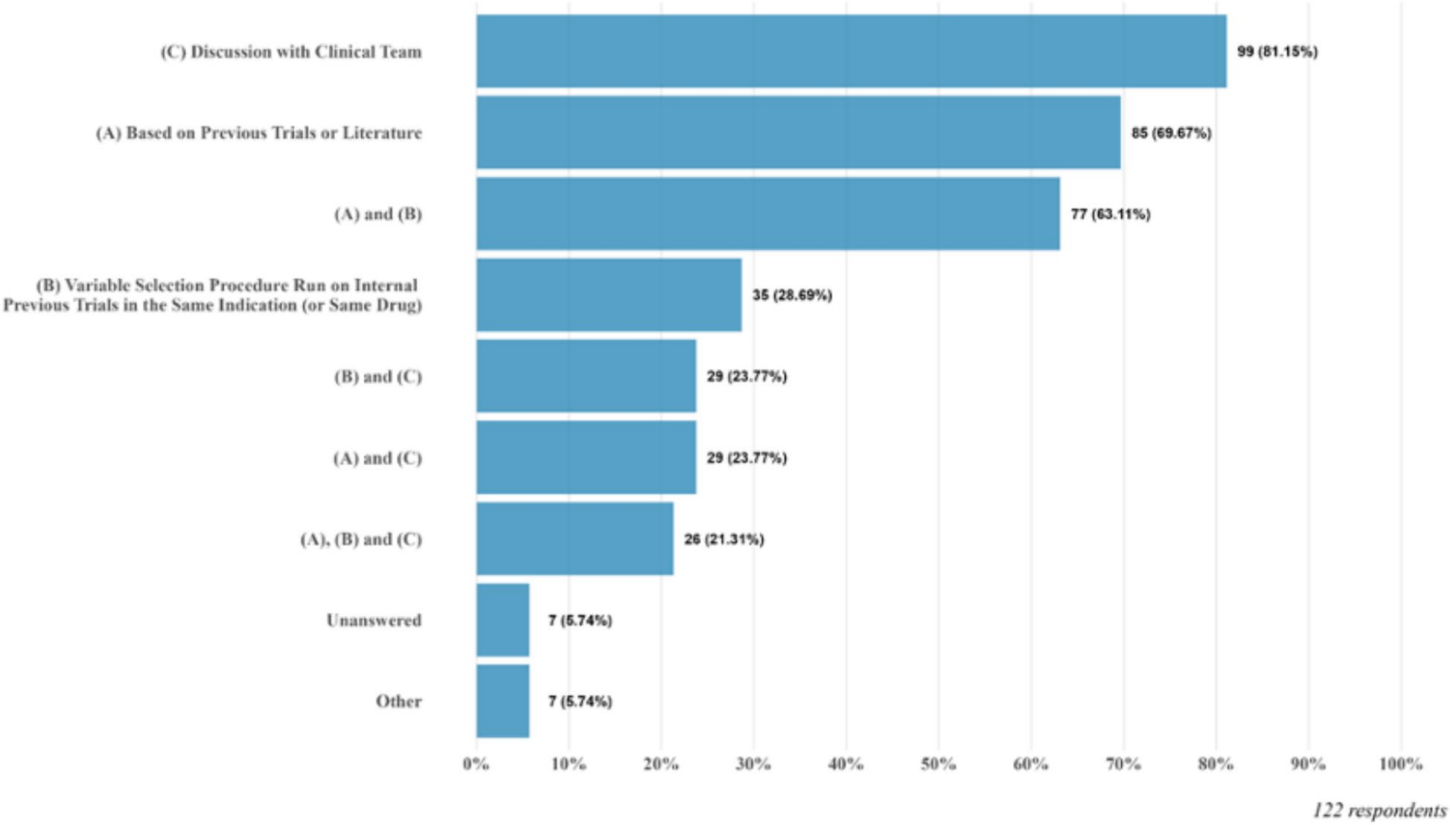
Response to Q9: How are the covariates for adjustment selected for the analysis model (if covariates beyond the stratification factors are used)

Given responses of Q7 and Q8, a subsequent question was asked in Q9 about how to select covariates for analysis beyond those used for stratification. The results are summarized in Fig. 2. Among the respondents to Q9, 81.2% of them prefer discussions with clinical teams, 69.7% of them use previous trials and the literature for references, and 28.7% of them utilize variable selection from available data sources. We also found out that 63.1% of respondents chose both options of using previous trials and the literature and variable selection from available data sources, 23.8% chose both options of discussions with clinical teams and using previous trials and the literature, and 23.8% chose both options of discussions with clinical teams and variable selection from available data sources. There are also 21.3% of respondents who selected all three options. From these results, we see a consistent trend on how one selects covariates for analysis, where the selection is mainly guided by medical knowledge and scientific rationales, which may be supplemented by data-driven approaches.

### Challenges with small strata (Q10-Q14)

When conducting a stratified analysis, a major concern arises with many strata, as this can result in sparse data within some of the strata, leading to unexpected and unstable estimates. To comprehensively understand the potential challenges and the common practice in managing small strata, we designed 5 specific questions (Q10 to Q14). Q10 aims to understand the potential consequences that may be caused by small strata. Among those responses, most of the concerns are focused on the following issues: 1) model does not converge (56.6%); 2) unstable estimate (e.g., the model estimates may change substantially with minor changes in the dataset; 45.9%); 3) high standard error and wide confidence interval of the estimate, which can lead to power loss (36.9%). Additionally, 16.4% of the respondents were concerned that the estimate is biased. It is important to clarify that there is no systematic bias in presence of small strata, and we will further discuss on this topic in Sect. 4. For a more detailed summary please refer to the Supplementary Material B, Figure S2.

Q11 and Q12 sought to gather opinions on defining “small strata” in practice. Q11 assumes a scenario in which the primary endpoint of interest is binary or continuous, so that the small strata are defined in terms of the number of subjects. According to the responses, less than 10 subjects in a stratum is considered as small by most respondents (44.3%); 27% of the respondents would consider less than 10 subjects in a stratum but they would consider less than 5 subjects in a stratum as small; 13.9% of the respondents would more conservatively consider a stratum as small if there are less than 15 subjects. Q12 focuses on the scenario when the study is planed with the time-to-event endpoint as the primary interest, and the small stratum is defined in terms of number of events. The responses are generally consistent with that of Q11; however, we also notice a slight growing proportion of respondents considering less than 5 events in a stratum as small. More detailed responses can be found in Supplementary Material B, Table S3.

**Fig. 3:**
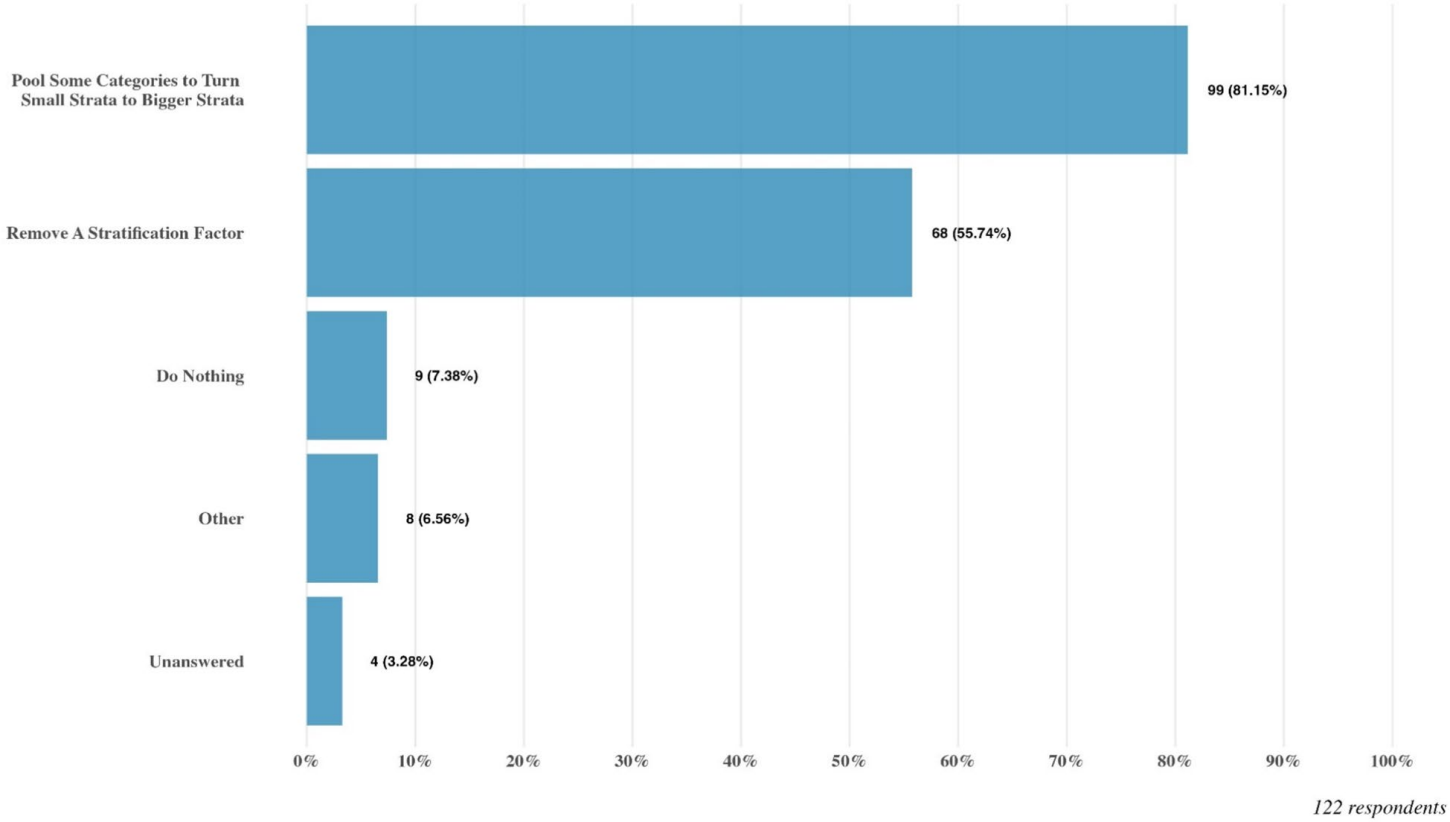
Response to Q13: If you have experienced challenges with small strata, how do you handle them in the analysis

Q13 and Q14 are two follow up questions exploring potential approaches to handle small strata. The most common approach selected by 81.1% of all respondents, is to pool some categories of the stratification factor to turn the small strata to bigger strata. Additionally, a notable proportion of respondents (55.7%) would take the strategy of removing a particular stratification factor. In response to Q14, most respondents (77%) would advocate for pre-specifying clear rules in protocol to handle the small stratum, while there are also 17.2% respondents think it is reasonable to take actions on an ad hoc basis. For more details, please refer to Fig. 3 and Table 2.

**Table 2:** Would the small stratum be handled by pre-specified rules or ad-hoc? Summary of results from Q14

Would the small stratum be handled by pre-specified rules or ad-hoc?	Answers
Pre-specify clear rules in protocol	77% (94/122)
On ad-hoc basis	17.2% (21/122)
Unanswered	5.7% (7/122)

### Regulatory interactions (Q16, Q17)

It is highly recommended to communicate and engage with health authorities early regarding covariate adjustment and stratification in the statistical model, especially for estimating conditional effects, as indicated in FDA guideline [1]: “*Sponsors should discuss with the relevant review divisions specific proposals in a protocol or statistical analysis plan containing nonlinear regression to estimate conditional treatment effects for the primary analysis.*” In this survey we collected participant’s experience on feedback from regulatory feedback on this topic via Q16 and Q17.

Q16 was designed to see whether sponsors had received generally consistent feedback from multiple regulatory agencies regarding covariate adjustment or stratified analysis, where 68 out of 108 respondents (63.0%) did not receive any feedback, suggesting a gap on health authority interaction. Among those 40 who had received consistent feedback from regulatory agencies (multiple choices allowed), about 70.0% (28/40) of the feedback are on too many strata or small strata, 67.5% (27/40) were asked to either combine strata or remove stratification factors that were used in stratified randomization. Regarding covariate adjusted analysis, 42.5% (17/40) are regarding including too many covariates in the analysis and 32.5% (13/40) are regarding the form of covariate variables. This is consistent with what is stated in the FDA guideline [1], where they require sponsors to discuss their proposal with the relevant review division if the number of covariates is large relative to the sample size or if proposing to adjust for a covariate with many levels (e.g., study sites).

Q17 was designed to collect the information on whether regulatory agencies have engaged with the participant in discussing covariate adjustment and stratified analyses. Among those 60 who had received any feedback from regulatory agencies (regardless of consistency), 68.3% (41/60) received feedback from US FDA, 41.7% (25/60) from EMA, 15.0% (9/60) from PMDA and 11.7% (7/60) from NMPA, and 26.7% (16/60) received feedback from regional agencies, including, Swissmedic, Health Canada, BfArM, and PEI.

### Current challenges (Q18, Q19)

We asked participants what challenges they faced in implementing covariate adjustment or stratified analysis in clinical trials (Q18). Participants were asked what resources or support would be most helpful for addressing these challenges (Q19).

**Fig. 4:**
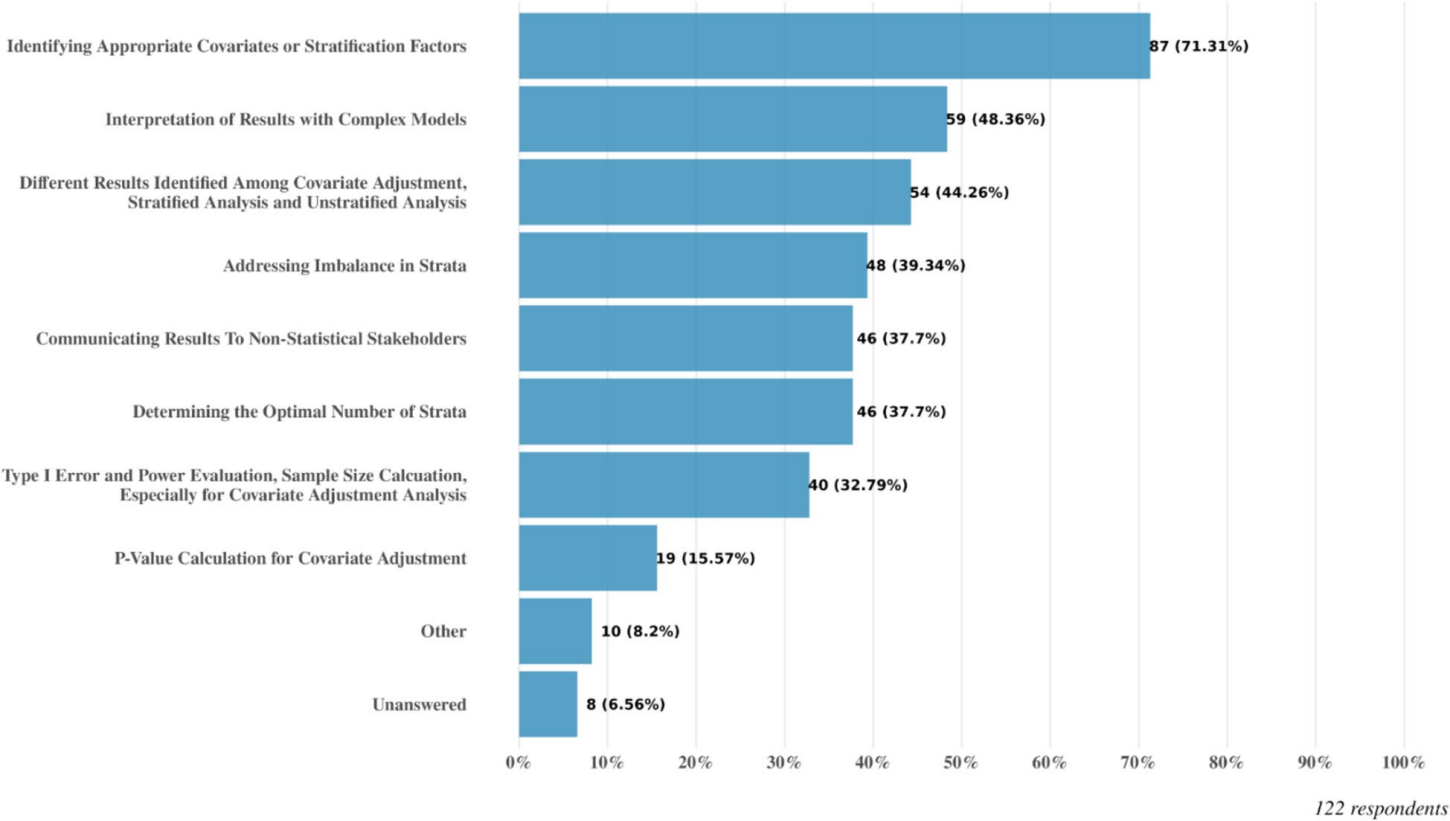
Response to Q18: In your experience, what are the most common challenges you have faced when implementing covariate adjustment or stratified analysis in clinical trials

Among the 122 responses, the majority (71.3%) faced the challenge of identifying appropriate covariates or stratification factors. Other challenges, ranging from 32.8% to 48.4% (Fig. 4), include: determining the optimal number of strata; addressing imbalance in strata; how to interpret different results identified among covariate adjustment, stratified analysis and unstratified analysis; interpretation of results with complex models; communicating results to non-statistical stakeholders; and type I error and power evaluation, sample size calculation, especially for covariate adjustment analysis.

To support addressing these challenges, most of the audience would like to gain resources or support via 1) accessing to comprehensive guidelines or best practices (76.2%); 2) case studies or examples from industry experts (68.9%); and 3) webinars or training sessions on specific topics (62.3%). Please refer to Supplementary Material B, Figure S3 for more details.

## Discussion

The results of our survey provided an important snapshot of the current practice on covariate adjustment and stratified analysis. In our sample of respondents, the majority (97 of the 122, 79.5%) were biostatisticians from pharmaceutical companies, mostly working in late-phase clinical trials. We gained many important insights from the input based on respondents’ experience.

### Conditional and unconditional treatment effects

One very interesting and surprising finding was the high proportion (around 60%) of the respondents who were unaware that a covariate adjusted or stratified model does not target the same estimand as an unadjusted or unstratified model in a non-linear model. This may result in large confusion in analysis results interpretation.

To clarify, it is important to emphasize that for non-linear models, including baseline covariates can change the interpretation of the treatment effect and the actual estimand targeted by the analysis. For example, the treatment effect parameter in a regression model without covariates targets the unconditional (overall average) treatment effect (estimand). However, including covariates in the same regression model changes the target estimand to a conditional treatment effect. Even without including covariate by treatment interaction, the unconditional and the conditional effects may differ due to the non-collapsibility of effect measures described in the FDA guidance [1]. In Table 1 of the guidance, an example of non-collapsibility of the odds ratio is illustrated for a hypothetical target population, which does not involve any confounding. A detailed discussion on the non-collapsibility of effect measures in regression models for binary and time-to-event outcomes is available in Daniel et al. [4], Morris et al. [2] and Wei et al. [5]. It is critical to distinguish this phenomenon from confounding bias. In a randomized trial, the randomization process ensures that baseline covariates are, on average, not associated with treatment assignment, and thus are not confounders in the classical sense, nor will there be any unmeasured confounders unaccounted if we were to choose to adjust for these in the model. Non-collapsibility, however, is a mathematical property of the effect measure itself (e.g., the odds ratio or hazard ratio) and will cause the magnitude of the conditional and marginal effects to diverge whenever the covariate is prognostic for the outcome, irrespective of randomization. The difference is therefore a feature of the chosen estimand, not a bias in the estimation. This is illustrated in Fig. 5 of Daniel et al. [4], the bottom right plot is an observational study with a confounder and the other panels represent randomized trials. In the randomized trial scenarios, the unconditional (unadjusted) and adjusted marginal estimate the same parameter for a marginal estimand. However, the adjusted marginal estimate provides better precision after adjustment for a prognostic factor (bottom left of Fig. 5 in Daniel et al. [4]). In the observational setting where the variable is considered a confounder (bottom-right of that same Figure), the unadjusted (unconditional) model would be a biased estimator of a marginal treatment effect compared to the adjusted marginal as they haven’t adjusted for the confounder in the unadjusted model.

When comparing different estimation methods (e.g., non-linear models with different covariates), one should be careful when evaluating “bias” or “efficiency gain” before the estimand is clearly defined; otherwise, it could be an "apple to orange" comparison as described by Daniel et al. [4]. In terms of the treatment effect estimation, stratified analysis for continuous and binary endpoint are often perceived as a weighted average of stratum-specific treatment effect. We wish to clarify that for a binary endpoint, only the risk difference can have a clearly defined estimand by averaging the stratum-specific risk difference [6]. The Cochran-Mantel–Haenszel (CMH) risk ratio and CMH odds ratio, which are common estimators, target a conditional estimand and they are not a weighted average of the stratum-specific treatment effect.

Currently, it in fact remains common practice in oncology to pre-specify the stratified Cox model as the primary analysis and the unstratified Cox model as a sensitivity analysis. An example of this approach is the GALLIUM study [7], discussed in Sun et al. [3]. It is generally expected that the hazard ratio estimates from the stratified and unstratified Cox models should be similar if the results are deemed "robust." However, this interpretation can be misleading. While the stratified Cox model estimates the conditional effect, the unstratified model captures the marginal effect. Due to the non-collapsibility of the hazard ratio, these two estimands may differ in magnitude, particularly when the stratification factors have strong prognostic influence [4]. Therefore, discrepancies between the stratified and unstratified hazard ratios may simply reflect the non-collapsibility of the estimands and not necessarily indicate a lack of robustness.

To address this, it is important at the study design stage to establish clear communication with health authorities regarding the roles of these analyses. Specifically, the unstratified Cox model can serve as a "supplementary analysis" [8] that targets a different Estimand—the marginal effect—than the stratified Cox model, which targets the conditional effect. Although the unstratified analysis provides an alternative perspective on the data, it is not expected to align with the stratified results because the two analyses are fundamentally estimating different estimands. At the analysis stage, if the stratified and unstratified hazard ratios differ substantially, it is critical to evaluate whether one or more stratification factors exhibit strong prognostic effects. This step is essential to distinguish whether the observed divergence in hazard ratio estimates arises from the non-collapsibility of the underlying estimands or if it indicates genuine issues with the robustness of the results.

### How to select covariates or stratification factors?

In terms of how to select/include covariates or stratification factors in the model, regulators, in general, encourage the inclusion of stratification factors in the statistical analysis. For example, ICH E9 [8] suggests that “*Factors on which randomization has been stratified should be accounted for later in the analysis*”. Similarly, FDA [1] also suggests “*A covariate adjustment model should generally include strata variables and can also include covariates not used for stratifying randomization*”. While EMA [9] has a similar view: *“… stratification variables, if not solely used for administrative reasons, should usually be included as covariates or stratification variables in the primary analysis regardless of their prognostic value*”, it emphasizes on justifiable rationales to include any additional covariates that were not used in the randomization: “*Any mismatch of non-administrative covariates between stratification and adjustment in the primary analysis must be explained and justified. This includes the use of covariates not stratified for in the randomization*.”

Based on the survey results, most of the respondents choose stratification factors and covariates under the guidance of medical knowledge and scientific rationales, which may be supplemented by data modelling approaches (e.g., machine learning) based on prior data. Recent examples of the latter include the PROCOVA approach [10], which predicts a prognostic score for the outcome under control based on a historical data set that is independent from the study data and then applying the prognostic score as covariate in an ANCOVA model for the actual data analysis of a clinical trial [11]. As emphasized in all above regulatory guidelines, pre-specification of how to adjust for stratification factors and additional covariates is important.

Statistical inference with stratified randomization needs special attention. Outcomes within strata are correlated and if ignored in analysis, the standard error for the treatment effect is biased upwards, leading to confidence interval that are too wide, type I error that is too low and reduction in power [12]. FDA [1] recommends that the standard error computation accounts for stratified randomization [13–15].

### How to deal with small strata?

If there are too many strata, the average sample size in each stratum will be small, simulations by [16] and [5] have shown that the stratified log-rank test exhibits a decrease in power under such scenarios. Power may even be inferior to the unstratified log-rank test despite strong prognostic effect of the adjusted stratification factor. Particularly, if certain strata have very small sample size, all subjects in a stratum may be assigned to the same treatment group, convergence issues are likely to arise. This is because the stratum-specific statistics may not be computed successfully or may yield infinite values. Additionally, there is a possibility that the treatment groups will not be balanced with respect to some prognostic factors. However, this imbalance is likely to be small and could equally favor either treatment group, thereby not introducing a systematic bias in the estimation of the treatment effect.

Based on many of our prior interactions with the regulators, discussions with regulators are facilitated by pre-specifying in the statistical analysis plan the list of covariates and the rule for combining strata. This also increases the statistical rigor and creditability of the analysis.

## Conclusions

This paper summarizes the results of a survey aiming to establish the current understanding of covariate adjusted, unstratified, and stratified analyses and their correspondence with the appropriate estimand. Recommendations are provided based on the results, addressing areas of consensus and gaps in knowledge for the application of estimands for conditional and unconditional treatment effects. This survey is the first of its kind on current practice of covariate adjustment in the context of estimands. However, the study has limitations. No formal study survey design methodology was implemented as this was intended as an exploratory and scoping exercise as a pilot survey. In addition to this, as a reviewer correctly highlighted, which we agree with in hindsight, some survey questions may not have been completely clear, thus potentially leading to some confusion. For example, a reviewer suggested that respondents may have differed in their interpretation of questions in relation to what an ideal study should be versus what they would have done in the past from a practical perspective. For example, in Question 10, a respondent may well know that it is best practice to pre-specify how small strata is handled. However, their response may instead be based on what they have done in previous studies, despite being aware of what the best practice is, or vice versa. Therefore, generally, there may be some unknown measurement error in terms of whether the responses reflect how respondents interpreted the questions as initially intended. The recommendations made by the Task Force based on the survey results do not follow a structured approach such as the Delphi method [17]. Future work could obtain a truly representative and comprehensive recommendation and guidance for covariate adjustment within estimands founded by a statistical stability of consensus. Due to resource constraints, the sample was selected based on the working group member’s networks, potentially leading to bias. Consequently, the sample may not reflect a true representative of researchers who may be applying and considering covariate adjustment in clinical trial practice. An additional potential bias is that individuals who did not respond to certain questions may have been unfamiliar with the query or may have lacked a response—this could inadvertently skew results towards those already acquainted with covariate adjustment. Keeping in line with our goal to maintain the utmost anonymity in our survey, we did not gather organization details, nor did we request information on the respondent's level of experience.

The survey evaluated common challenges when implementing covariate adjustment or stratified analysis in clinical trials. Multiple agencies have provided guidance, and there are literatures (e.g. Wei et al. [5] and Van Lancker et al. [18]) and workshops focusing on best practices (e.g. EFSPI and ASA workshops). Additionally, there are working groups and teams working on software development to address these challenges. The survey did not ask questions about adjusting for covariates when using the difference in restricted mean survival time for time to event outcomes with censoring. In contrast to the hazard ratio, this measure is collapsible and covariate-adjustment can still estimate the same estimand, thus eliminating the confusion we observed between interpreting the unadjusted and adjusted hazard ratio.

In summary, this paper presents the findings of a survey aimed at understanding the current practices and challenges related to covariate adjustment in the context of estimands. While the survey provides valuable insights, it is important to consider its limitations and the potential for bias in the sample selection. The survey also highlights the need for comprehensive recommendations and guidance, as well as the availability of resources and working groups to address the challenges identified in implementing covariate adjustment and stratified analysis in clinical trials.

## Supplementary Information

Supplementary Material 1.

Supplementary Material 2.

## Data Availability

The full survey report results are available in the supplementary material (Supplementary Material C).
