# Supplementary material for: Current practice on covariate adjustment and stratified analysis —based on survey results by ASA oncology estimand working group conditional and marginal effect task force
Source: BMC Med Res Methodol. 2025 Nov 4;25:249. doi: 10.1186/s12874-025-02670-7 (PMC12584542; doi:10.1186/s12874-025-02670-7)
Supplement: Supplementary file 1 — Supplementary Material 1. [file 12874_2025_2670_MOESM1_ESM.docx]

# Supplementary Material A: Survey Form


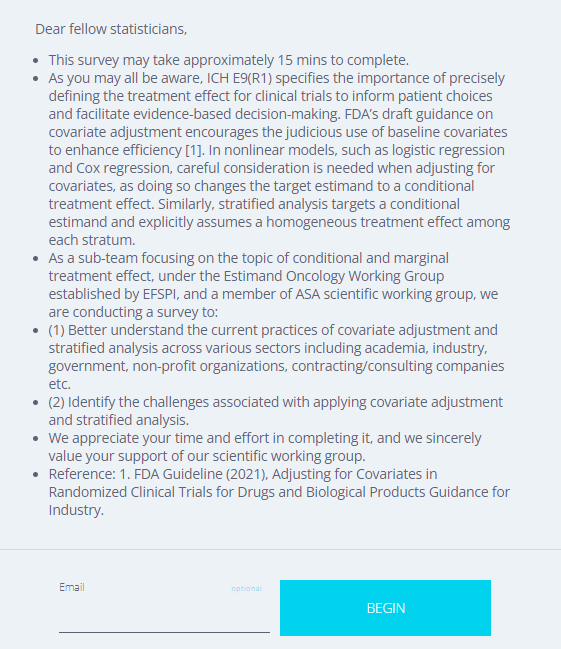


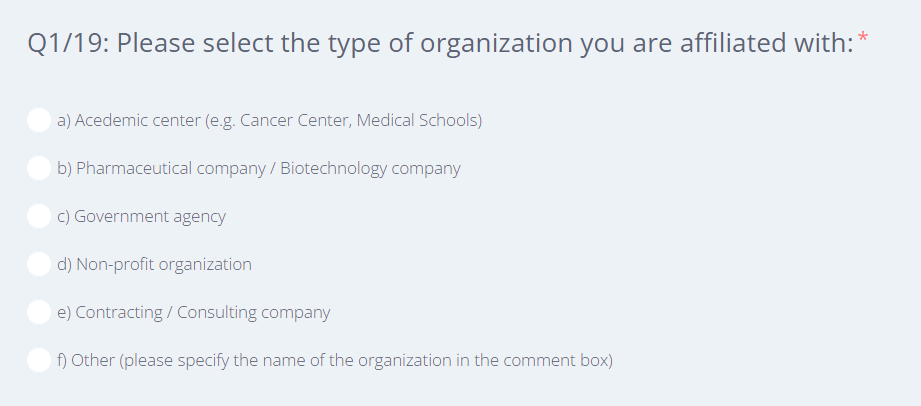


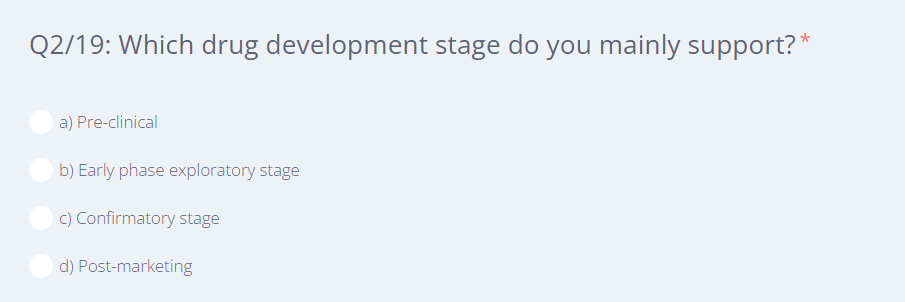


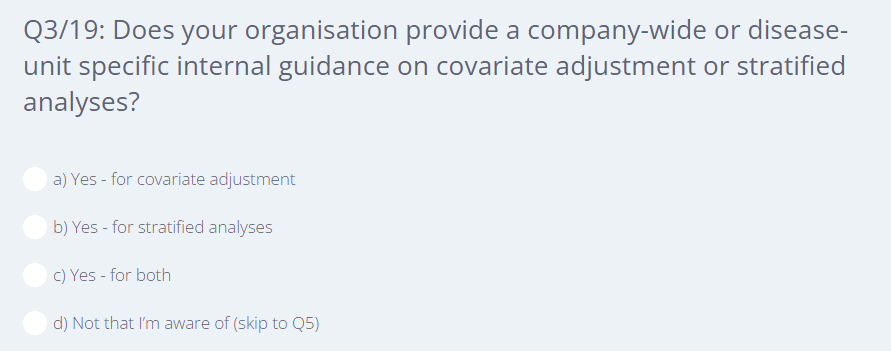


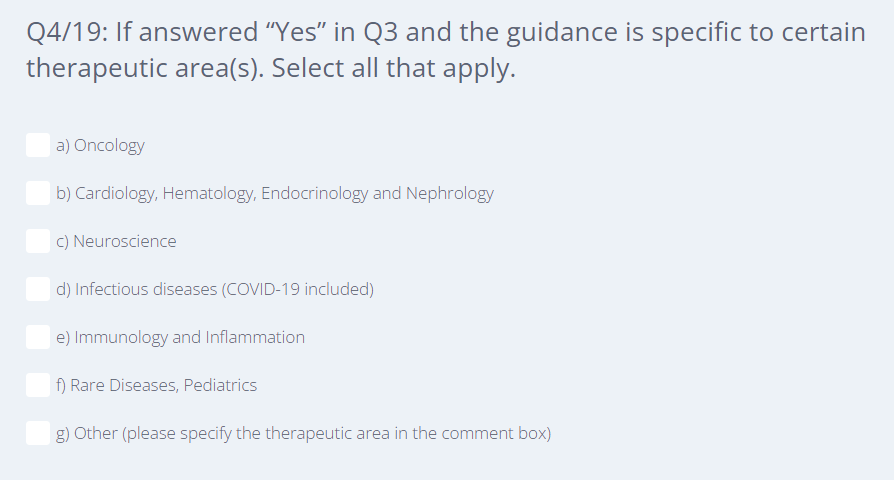


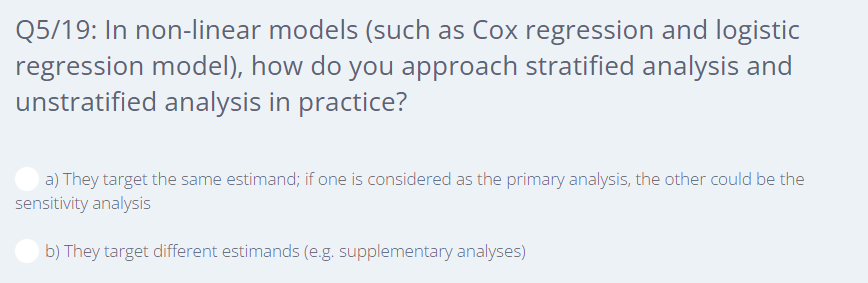


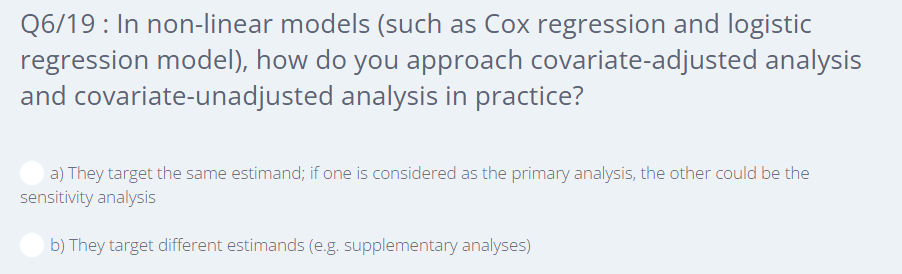


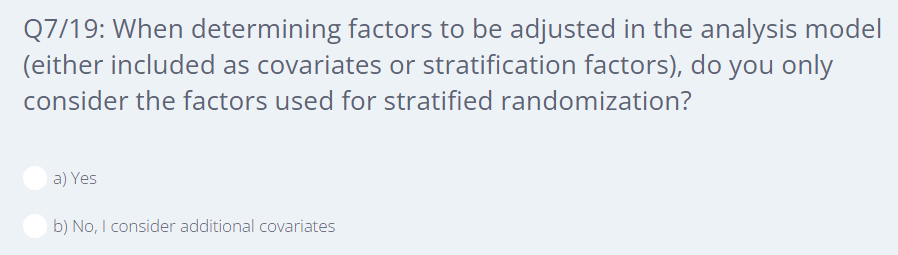


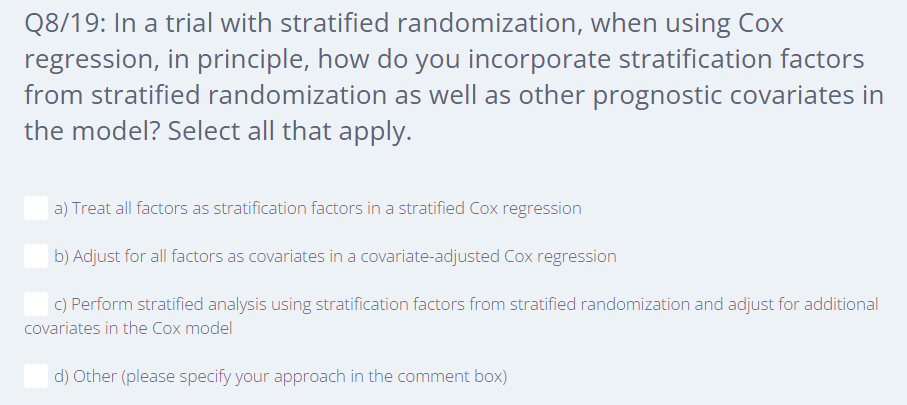


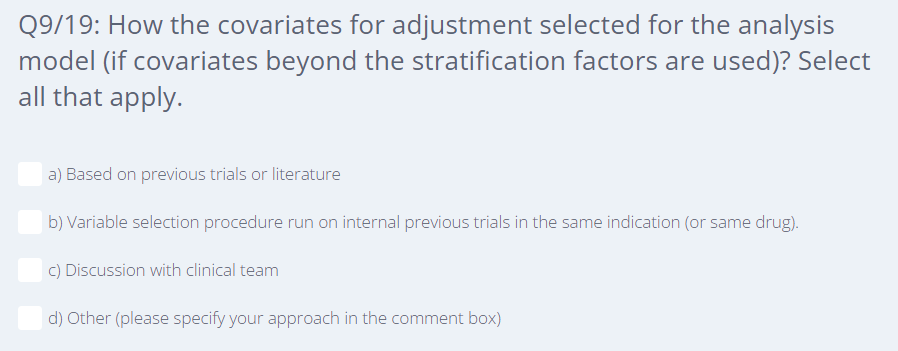


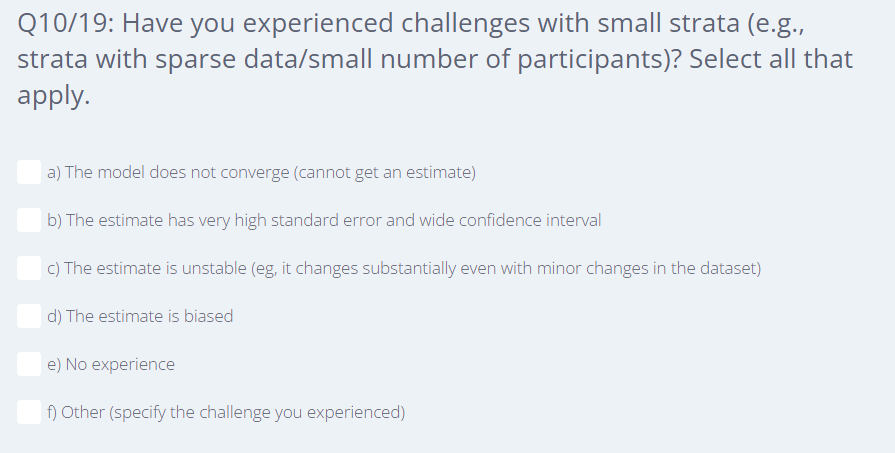


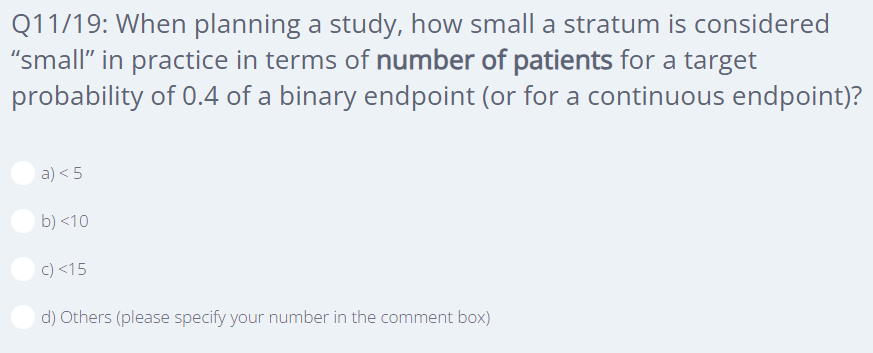


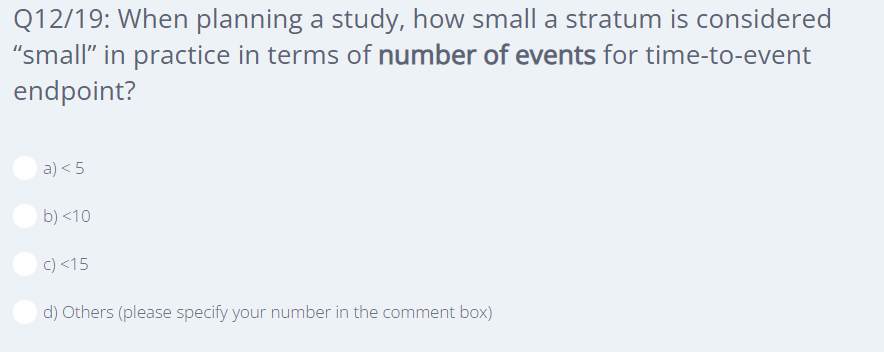


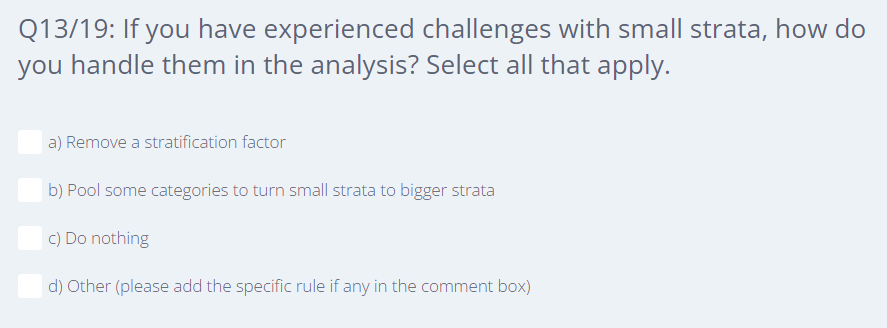


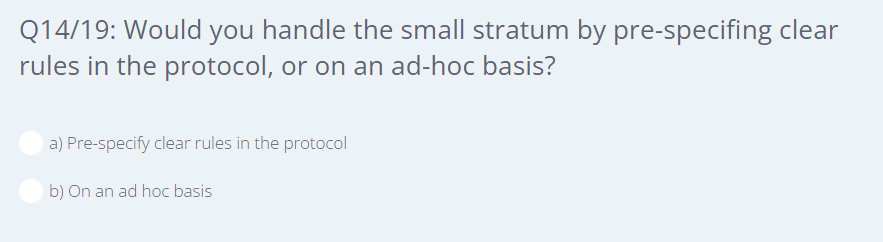


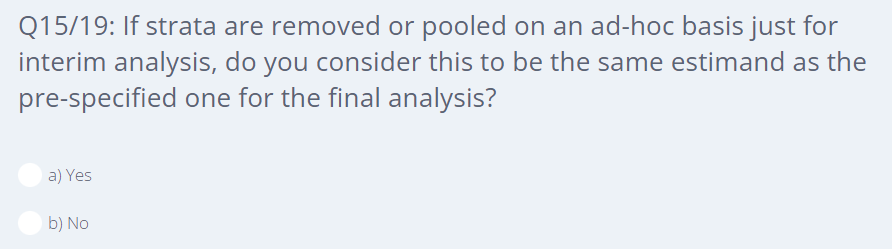


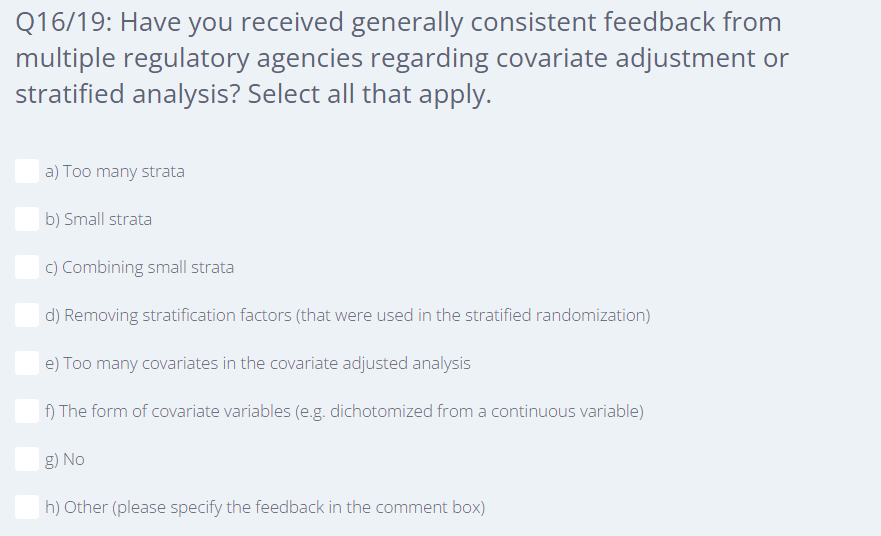


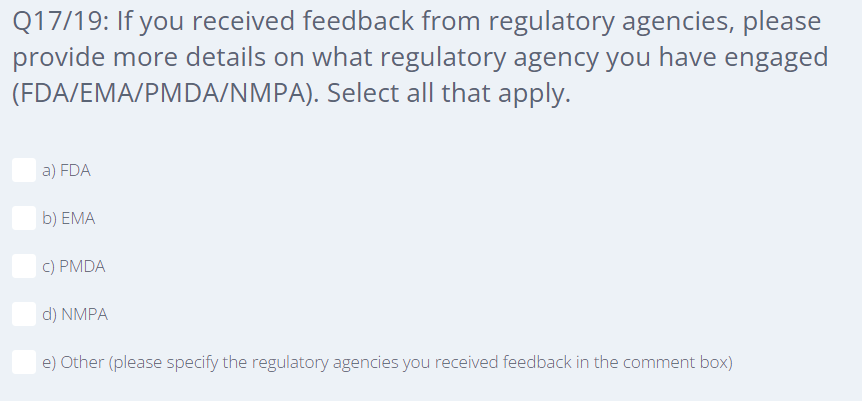


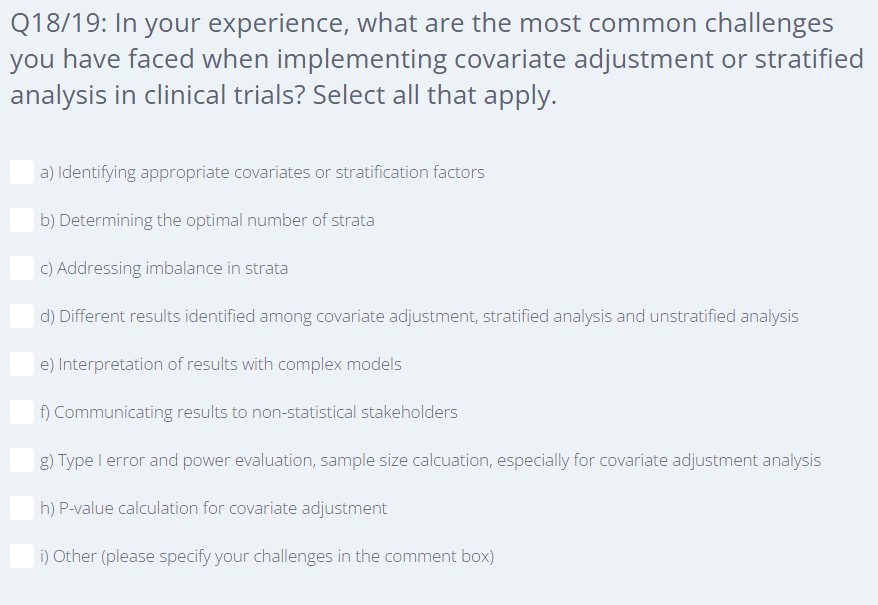


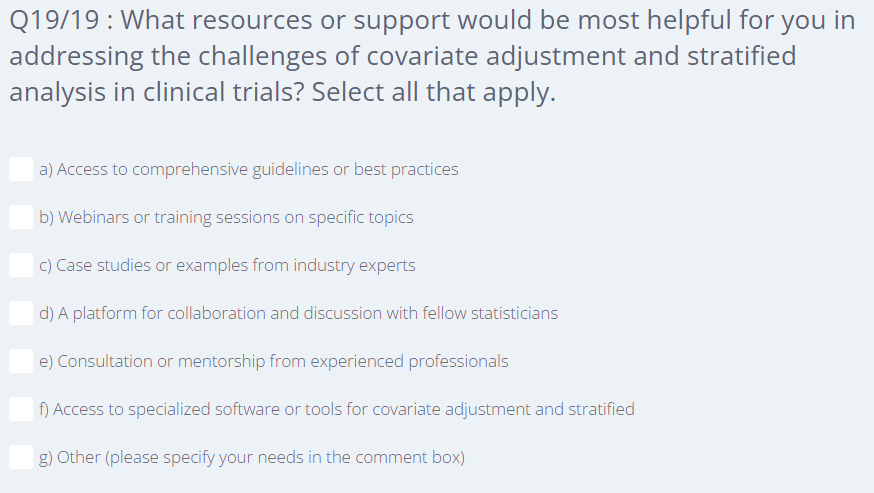


# Supplementary Material B: Summaries of Survey Results

**Table S1**:    Characteristics of the respondents. Summary of results from Q1 and Q2.

|  | Respondents (N = 122) |
| --- | --- |
| Country |  |
| United States | 57 (46.7%) |
| China (Including HK) | 24 (19.7%) |
| Switzerland | 14 (11.5%) |
| Other or unknown | 26 (21.3%) |
| Affiliates |  |
| Pharmaceutical / Biotechnology company | 97 (79.5%) |
| Contracting / Consulting company | 11 (9.0%) |
| Acedemic centre | 10 (8.2%) |
| Government agency | 3(2.5%) |
| Non-profit organization | 1 (0.8%) |
| Stage of development |  |
| Confirmatory | 100 (81.3%) |
| Early phase exploratory | 22 (17.9%) |
| Pre-Clinical | 1 (0.8%) |

**Table S2**:  Availability of internal guidelines on covariate adjustment or stratified analyses. Summary of results from Q3.

| **Company-wise guidance** | **Number of Respondents (N = 122)** |
| --- | --- |
| Not aware of | 72 (59.0%) |
| Provided | 49 (40.2%) |
| Both stratified randomization and covariate adjustment | 30 (24.6%) |
| Stratified randomization only | 11 (9.0%) |
| Covariate adjustment only | 8 (6.6%) |

**Table S3**: How small a stratum is considered “small” in practice? Summary of results from Q11, Q12.

| How small a stratum is considered as “small” | For a study powered for binary endpoint | For a study powered for time-to-event endpoint |
| --- | --- | --- |
| <5 (patients/events) | 27% (33/122) | 33.6% (41/122) |
| <10 (patients/events) | 44.3% (54/122) | 34.4% (42/122) |
| <15 (patients/events) | 13.9% (17/122) | 15.6% (19/122) |
| Unanswered | 5.7% (7/122) | 8.2% (10/122) |


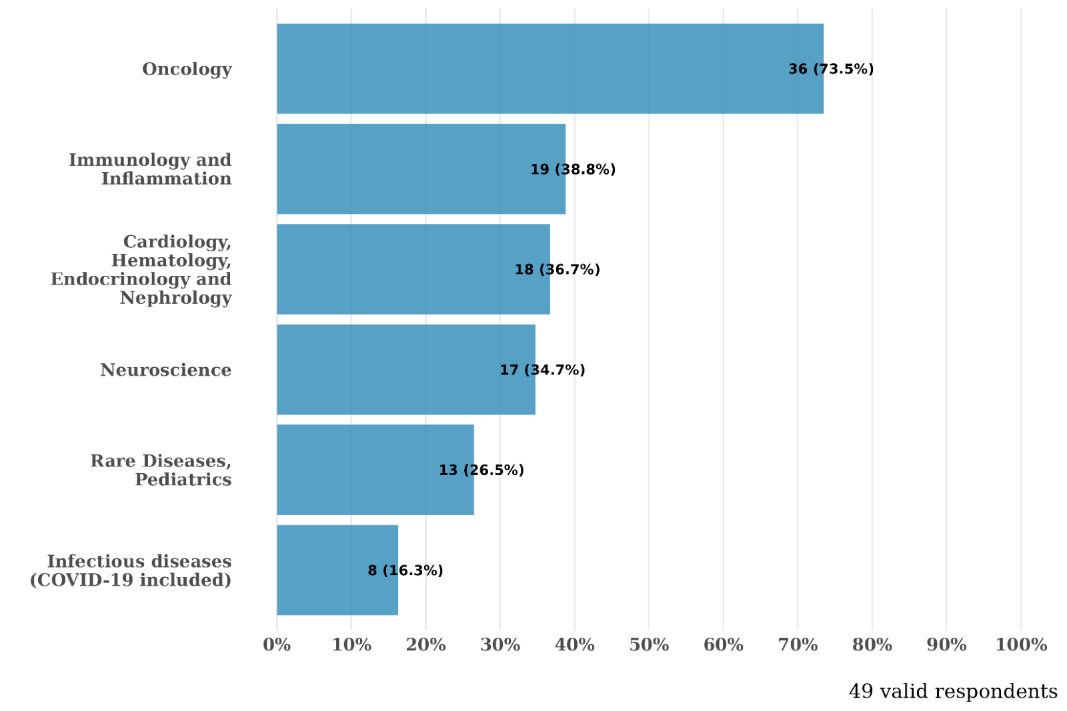


**Figure S1.** Therapeutic areas covered by the internal guidelines on covariate adjustment or stratified analyses. Summary of results from Q4.


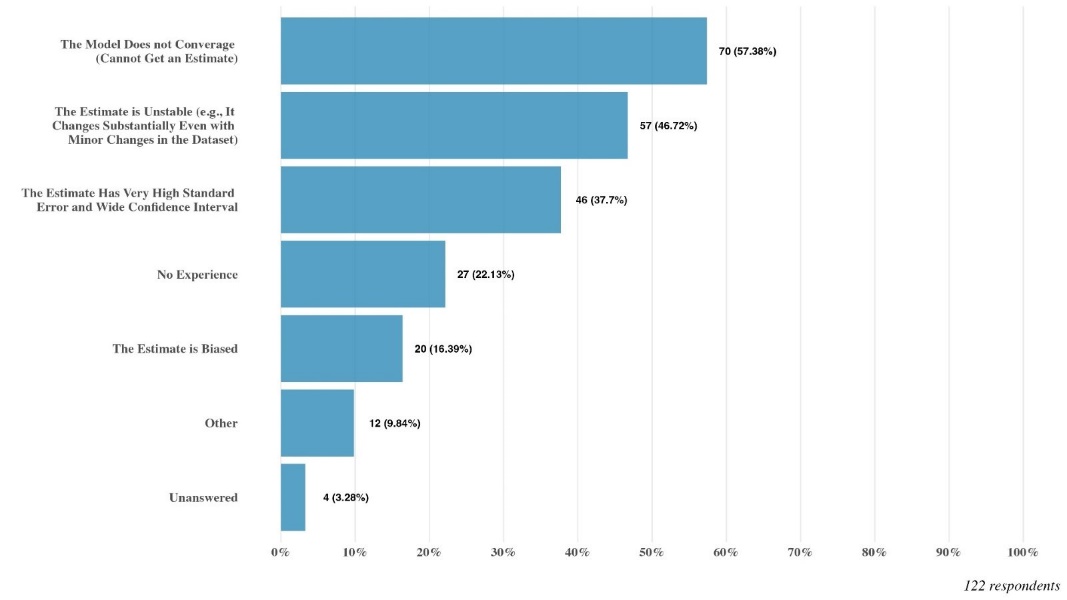


**Figure S2.** Response to Q10: Have you experienced challenges with small strata (e.g., strata with sparse data/small number of participants)?


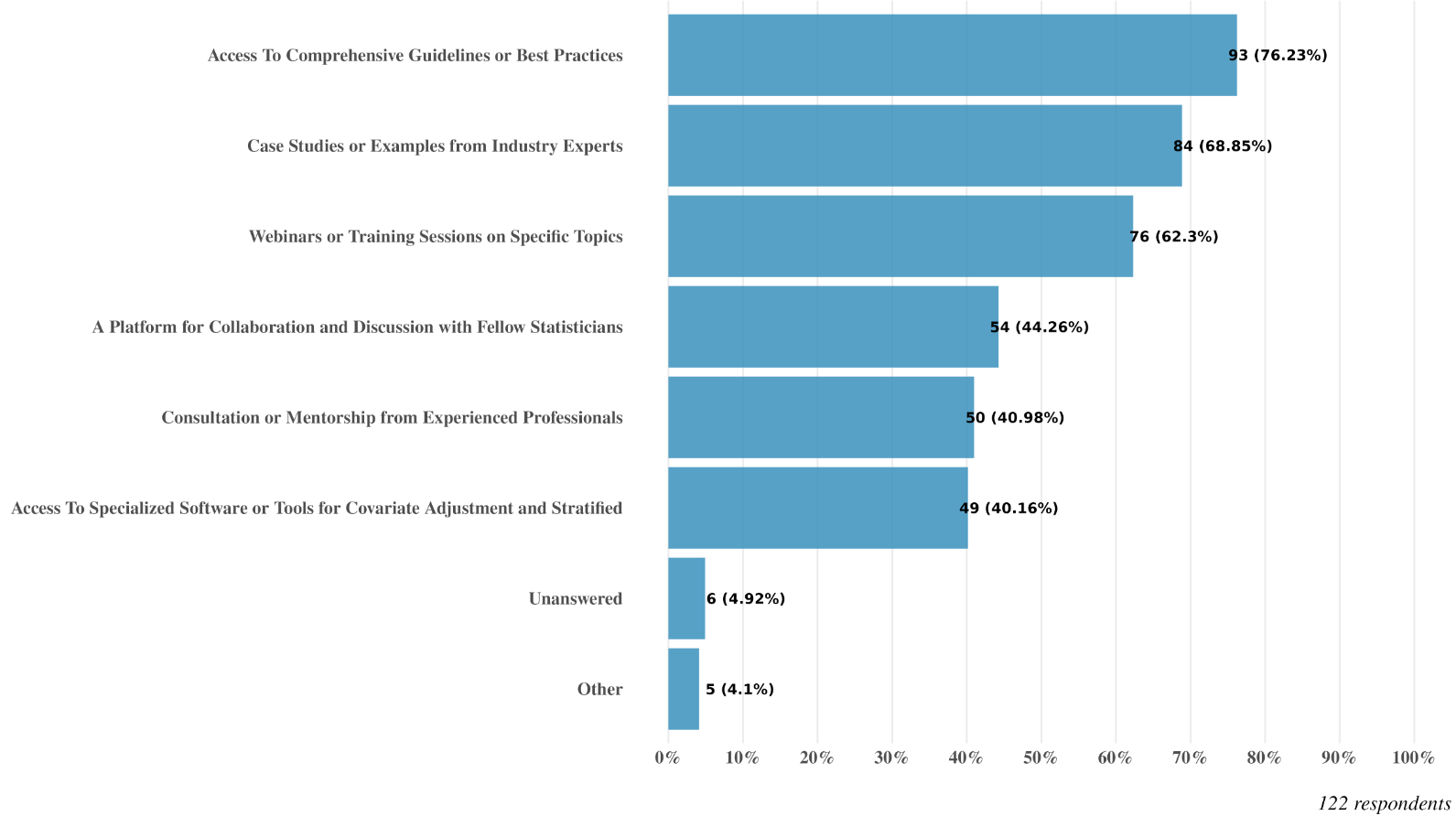


**Figure S3.** Response to Q19: What resources or support would be most helpful for you in addressing the challenges of covariate adjustment and stratified analysis in clinical trials?
