# Supplementary material for: Current practice on covariate adjustment and stratified analysis —based on survey results by ASA oncology estimand working group conditional and marginal effect task force
Source: BMC Med Res Methodol. 2025 Nov 4;25:249. doi: 10.1186/s12874-025-02670-7 (PMC12584542; doi:10.1186/s12874-025-02670-7)
Supplement: Supplementary file 2 — Supplementary Material 2. [file 12874_2025_2670_MOESM2_ESM.pdf]

Survey on current practice of covariate adjustment and stratified analysis

Dear fellow statisticians,

- This survey may take approximately 15 mins to complete.
- As you may all be aware, ICH E9(R1) specifies the importance of precisely defining the treatment effect for clinical trials to inform patient choices and facilitate evidence-based decision-making. FDA's draft guidance on covariate adjustment encourages the judicious use of baseline covariates to enhance efficiency [1]. In nonlinear models, such as logistic regression and Cox regression, careful consideration is needed when adjusting for covariates, as doing so changes the target estimand to a conditional treatment effect. Similarly, stratified analysis targets a conditional estimand and explicitly assumes a homogeneous treatment effect among each stratum.
- As a sub-team focusing on the topic of conditional and marginal treatment effect, under the Estimand Oncology Working Group established by EFSPI, and a member of ASA scientific working group, we are conducting a survey to:
- (1) Better understand the current practices of covariate adjustment and stratified analysis across various sectors including academia, industry, government, non-profit organizations, contracting/ consulting companies etc.
- (2) Identify the challenges associated with applying covariate adjustment and stratified analysis.
- We appreciate your time and effort in completing it, and we sincerely value your support of our scientific working group.
- Reference: 1. FDA Guideline (2021), Adjusting for Covariates in Randomized Clinical Trials for Drugs and Biological Products Guidance for Industry.

Q1 Q1/19: Please select the type of organization you are affiliated with:*

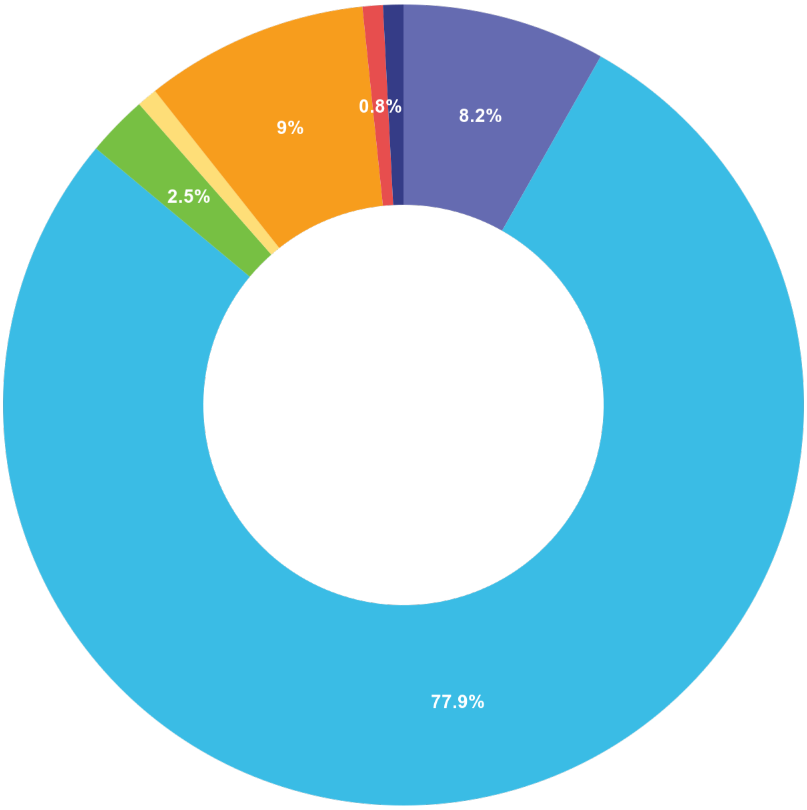

Answered: 122 **Unanswered:** 0

	Choice	Total
	a) Acedemic center (e.g. Cancer Center, Medical Schools)	10
	b) Pharmaceutical company / Biotechnology company	95
	c) Government agency	3
	d) Non-profit organization	1
	e) Contracting / Consulting company	11
	f) Other (please specify the name of the organization in the comment box)	1
	__archived__	1

Q2 Q2/19: Which drug development stage do you mainly support?*

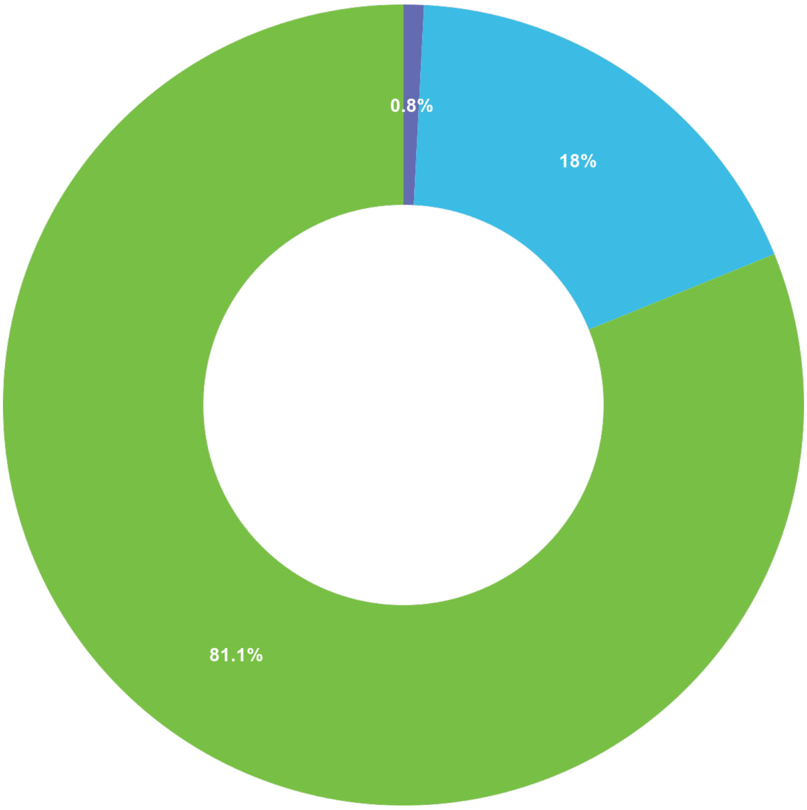

Answered: 122 **Unanswered:** 0

	Choice	Total
	a) Pre-clinical	1
	b) Early phase exploratory stage	22
	c) Confirmatory stage	99
	d) Post-marketing	0

Q3 Q3/19: Does your organisation provide a company-wide or disease-unit specific internal guidance on covariate adjustment or stratified analyses?

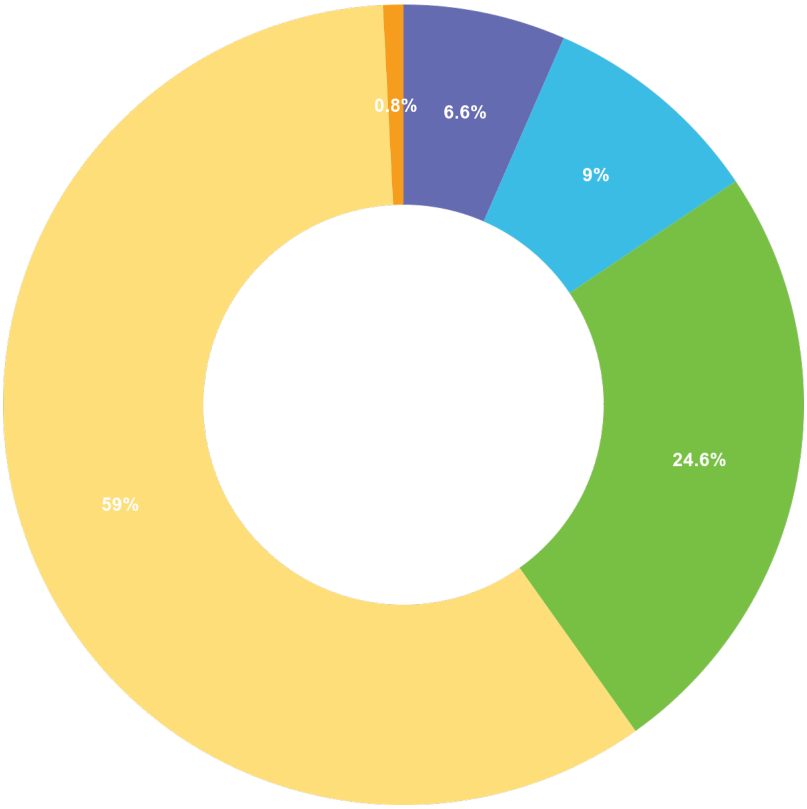

Answered: 122 **Unanswered:** 0

	Choice	Total
	a) Yes - for covariate adjustment	8
	b) Yes - for stratified analyses	11
	c) Yes - for both	30
	d) Not that I'm aware of (skip to Q5)	72
	__archived__	1

Q4 Q4/19: If answered "Yes" in Q3 and the guidance is specific to certain therapeutic area(s). Select all that apply.

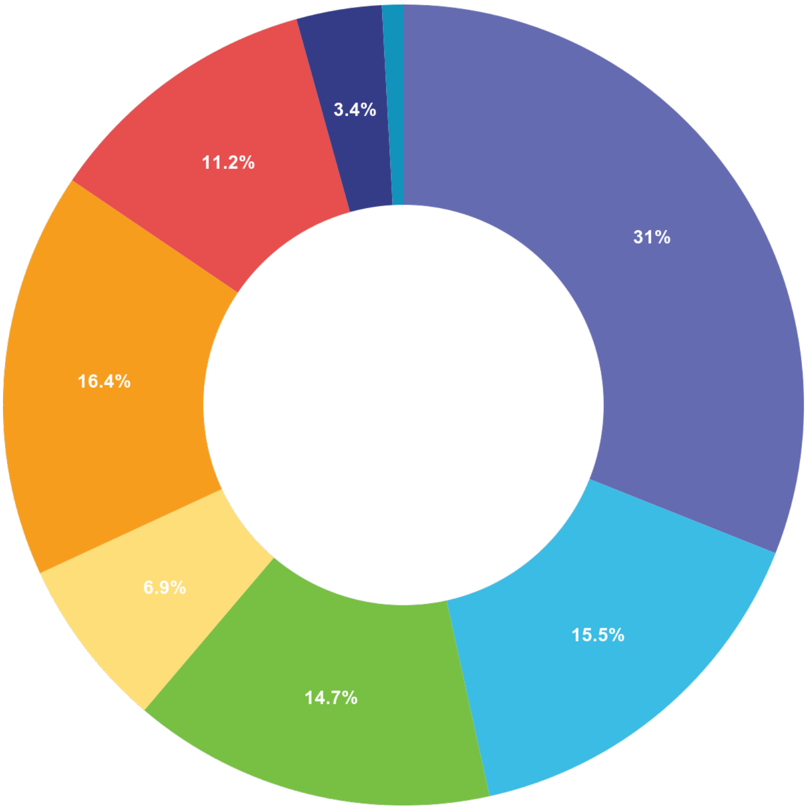

Answered: 50 **Unanswered:** 72

	Choice	Total
	a) Oncology	36
	b) Cardiology, Hematology, Endocrinology and Nephrology	18
	c) Neuroscience	17
	d) Infectious diseases (COVID-19 included)	8
	e) Immunology and Inflammation	19
	f) Rare Diseases, Pediatrics	13
	g) Other (please specify the therapeutic area in the comment box)	4
	__archived__	1

Q5 Q5/19: In non-linear models (such as Cox regression and logistic regression

model), how do you approach stratified analysis and unstratified analysis in practice?

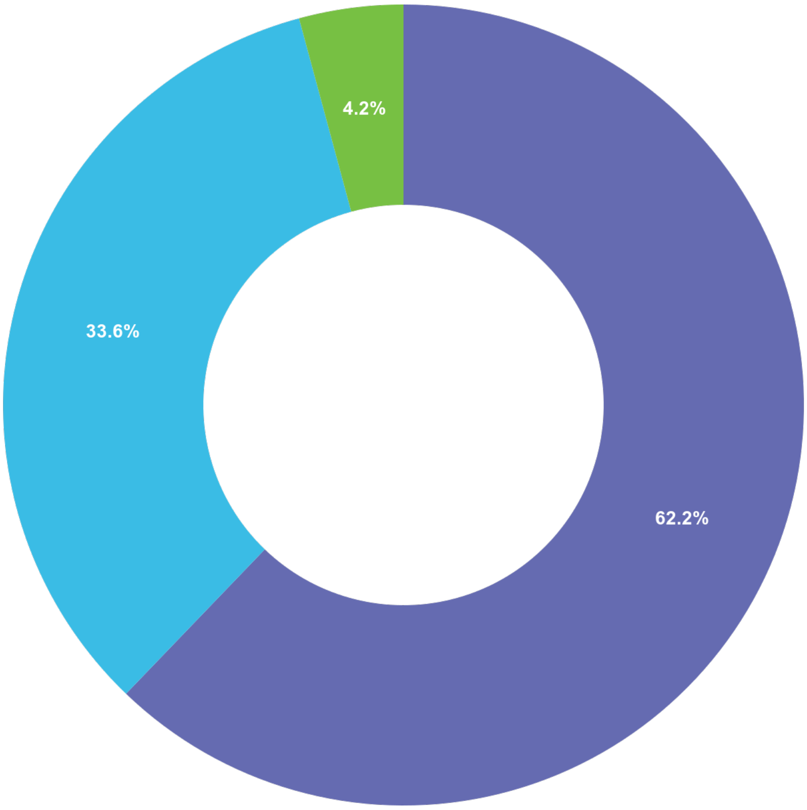

Answered: 119 **Unanswered:** 3

	Choice	Total
	a) They target the same estimand; if one is considered as the primary analysis, the other could be the sensitivity analysis	74
	b) They target different estimands (e.g. supplementary analyses)	40
	__archived__	5

Q6 Q6/19 : In non-linear models (such as Cox regression and logistic regression

model), how do you approach covariate-adjusted analysis and covariate-unadjusted analysis in practice?

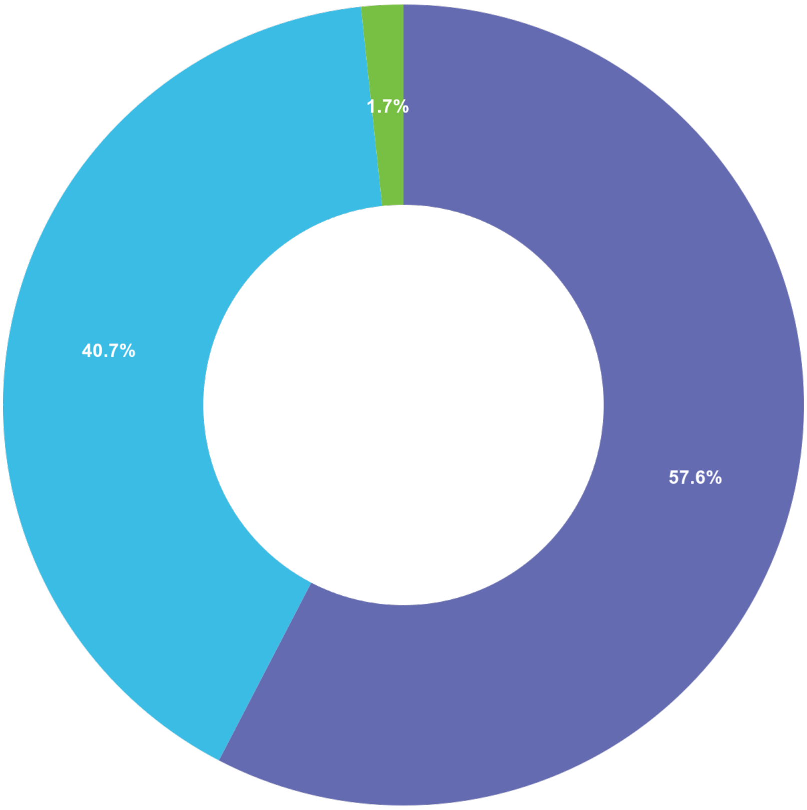

Answered: 118 **Unanswered:** 4

Choice	Total
a) They target the same estimand; if one is considered as the primary analysis, the other could be the sensitivity analysis	68
b) They target different estimands (e.g. supplementary analyses)	48
__archived__	2

Q7 Q7/19: When determining factors to be adjusted in the analysis model (either included as covariates or stratification factors), do you only consider the factors used for stratified randomization?

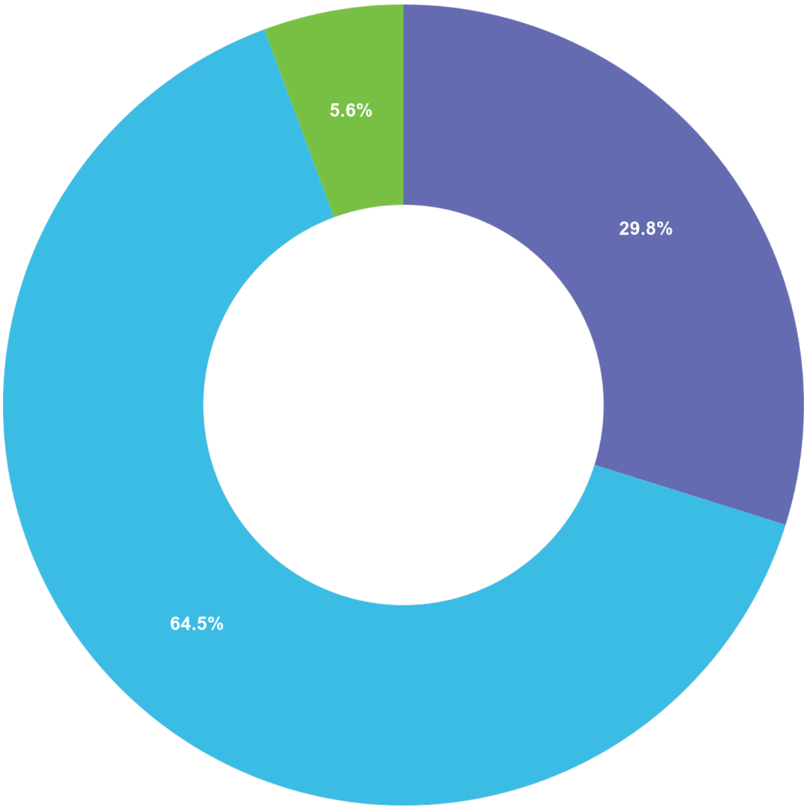

Answered: 121 **Unanswered:** 1

	Choice	Total
	a) Yes	37
	b) No, I consider additional covariates	80
	__archived__	7

Q8 Q8/19: In a trial with stratified randomization, when using Cox regression, in principle, how do you incorporate stratification factors from stratified randomization as well as other prognostic covariates in the model? Select all that apply.

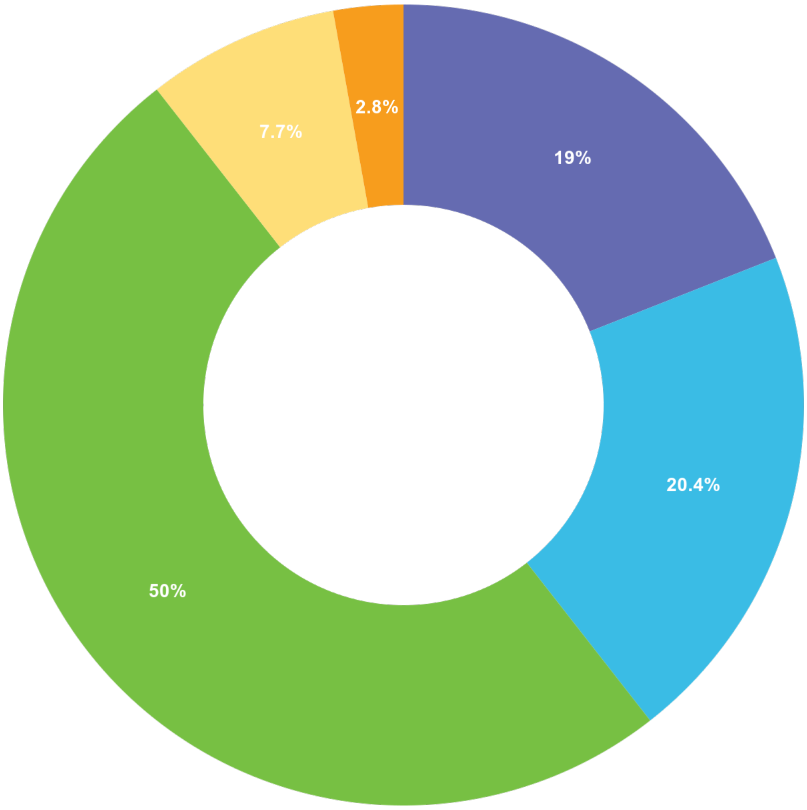

Answered: 114 **Unanswered:** 8

	Choice	Total
●	a) Treat all factors as stratification factors in a stratified Cox regression	27
●	b) Adjust for all factors as covariates in a covariate-adjusted Cox regression	29
●	c) Perform stratified analysis using stratification factors from stratified randomization and adjust for additional covariates in the Cox model	71
●	d) Other (please specify your approach in the comment box)	11
●	__archived__	4

Q9 Q9/19: How the covariates for adjustment selected for the analysis model (if covariates beyond the stratification factors are used)? Select all that apply.

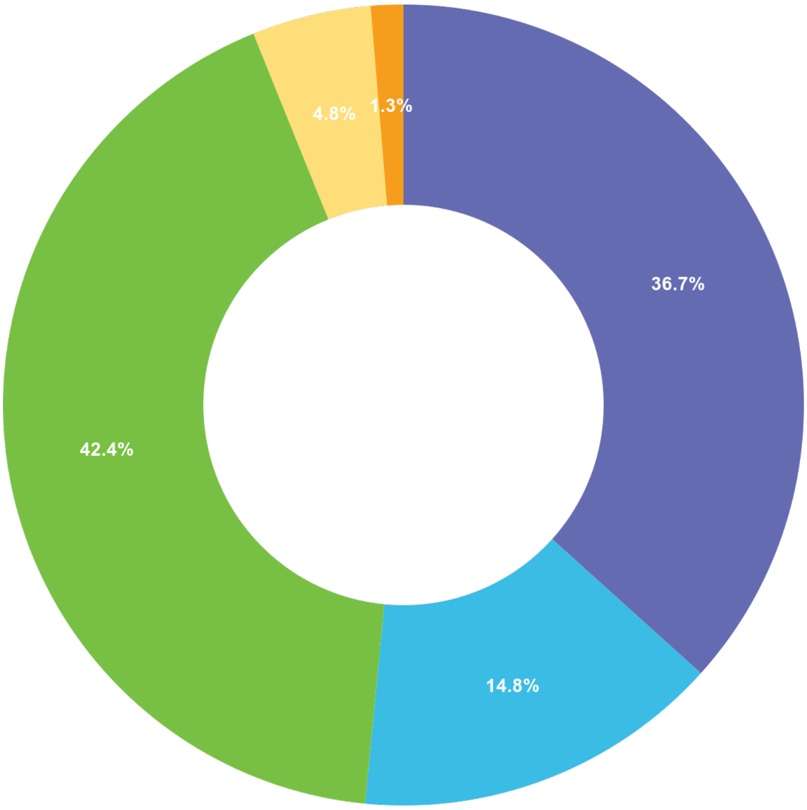

Answered: 115 **Unanswered:** 7

Choice	Total
a) Based on previous trials or literature	84
b) Variable selection procedure run on internal previous trials in the same indication (or same drug).	34
c) Discussion with clinical team	97
d) Other (please specify your approach in the comment box)	11
__archived__	3

Q10 Q10/19: Have you experienced challenges with small strata (e.g., strata with sparse data/small number of participants)? Select all that apply.

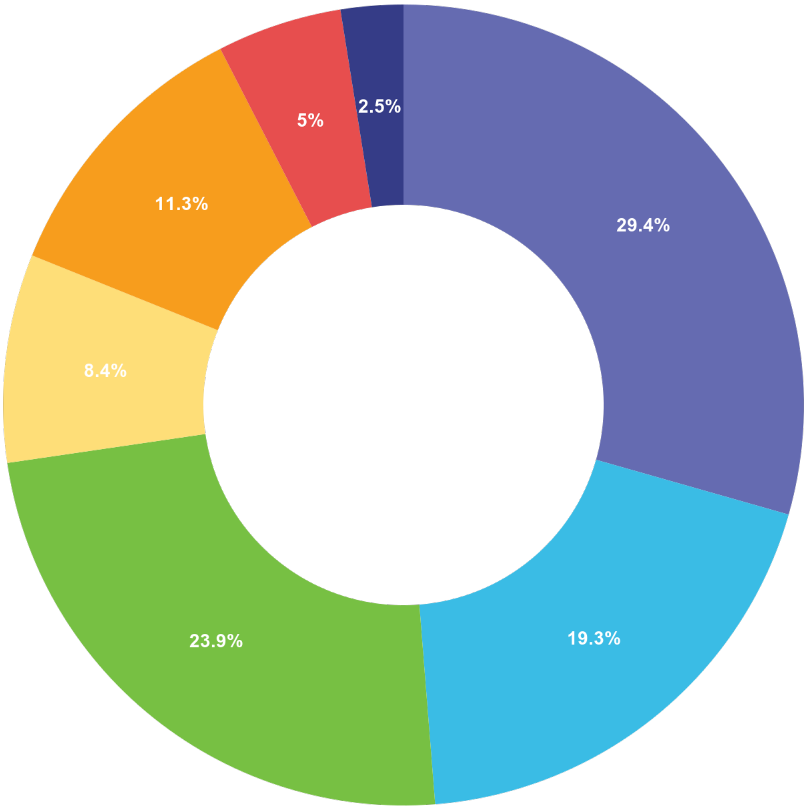

Answered: 118 **Unanswered:** 4

Choice	Total
a) The model does not converge (cannot get an estimate)	70
b) The estimate has very high standard error and wide confidence interval	46
c) The estimate is unstable (eg, it changes substantially even with minor changes in the dataset)	57
d) The estimate is biased	20
e) No experience	27
f) Other (specify the challenge you experienced)	12
__archived__	6

Q11 Q11/19: When planning a study, how small a stratum is considered “small” in practice in terms of **number of patients** for a target probability of 0.4 of a binary endpoint (or for a continuous endpoint)?

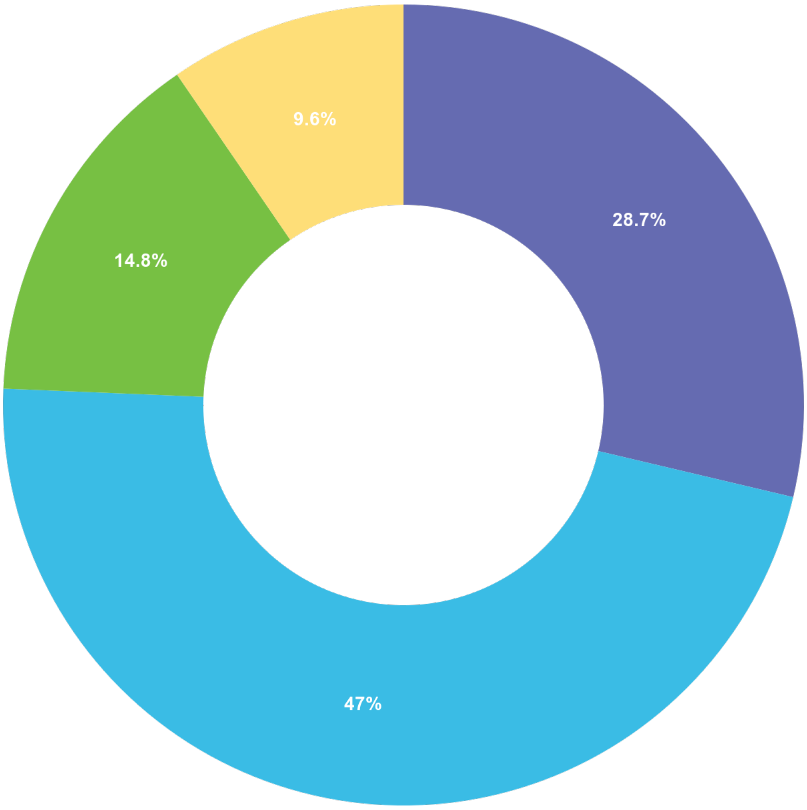

Answered: 115 **Unanswered:** 7

	Choice	Total
	a) < 5	33
	b) <10	54
	c) <15	17
	d) Others (please specify your number in the comment box)	11

Q12 Q12/19: When planning a study, how small a stratum is considered “small” in practice in terms of **number of events** for time-to-event endpoint?

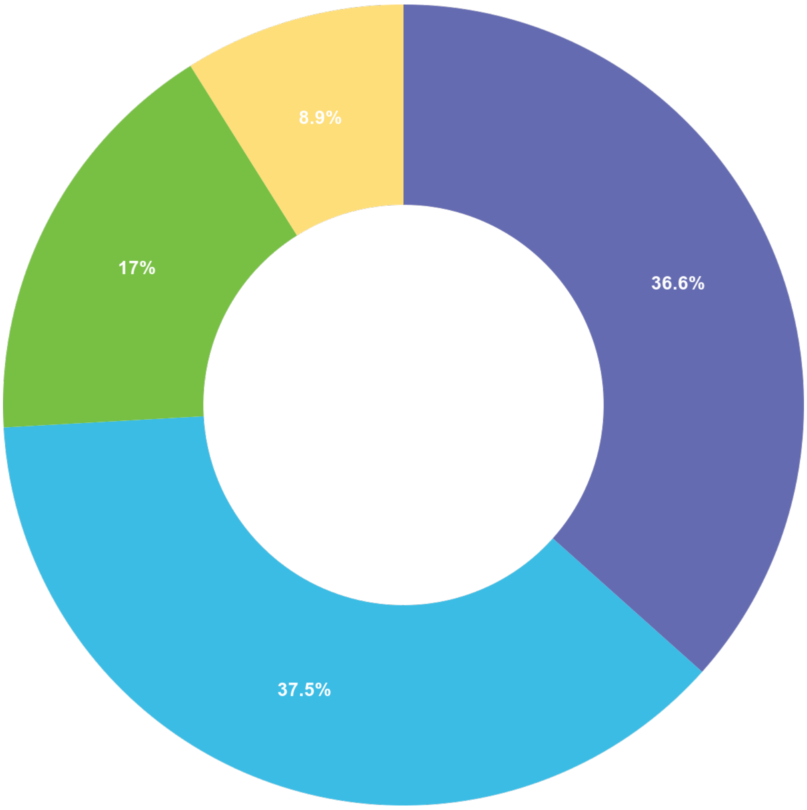

Answered: 112 **Unanswered:** 10

	Choice	Total
a)	< 5	41
b)	<10	42
c)	<15	19
d)	Others (please specify your number in the comment box)	10

Q13 Q13/19: If you have experienced challenges with small strata, how do you handle them in the analysis? Select all that apply.

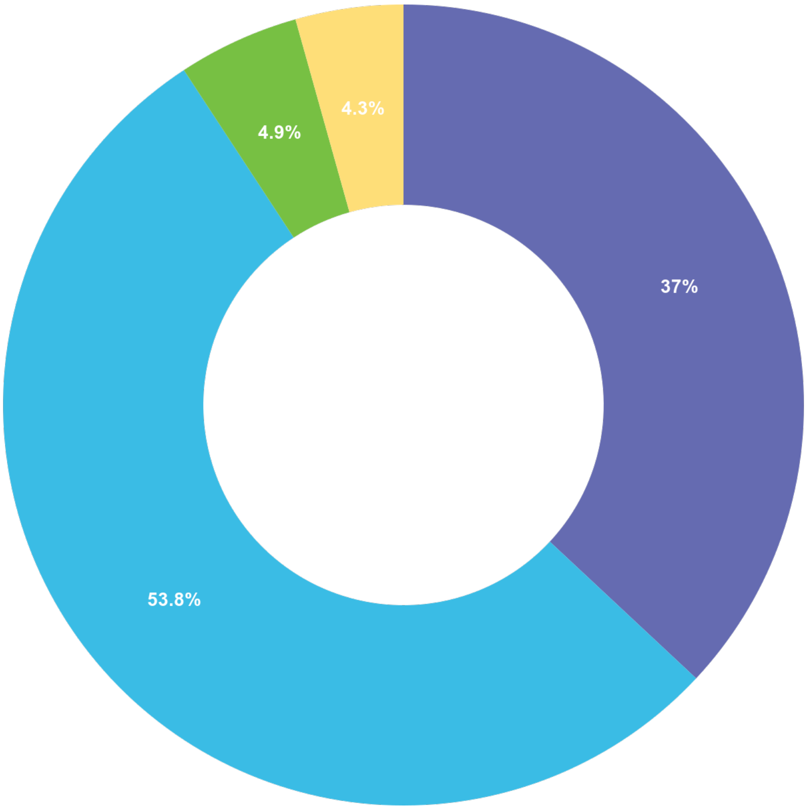

Answered: 118 **Unanswered:** 4

	Choice	Total
●	a) Remove a stratification factor	68
●	b) Pool some categories to turn small strata to bigger strata	99
●	c) Do nothing	9
●	d) Other (please add the specific rule if any in the comment box)	8

Q14 Q14/19: Would you handle the small stratum by pre-specifing clear rules in the protocol, or on an ad-hoc basis?

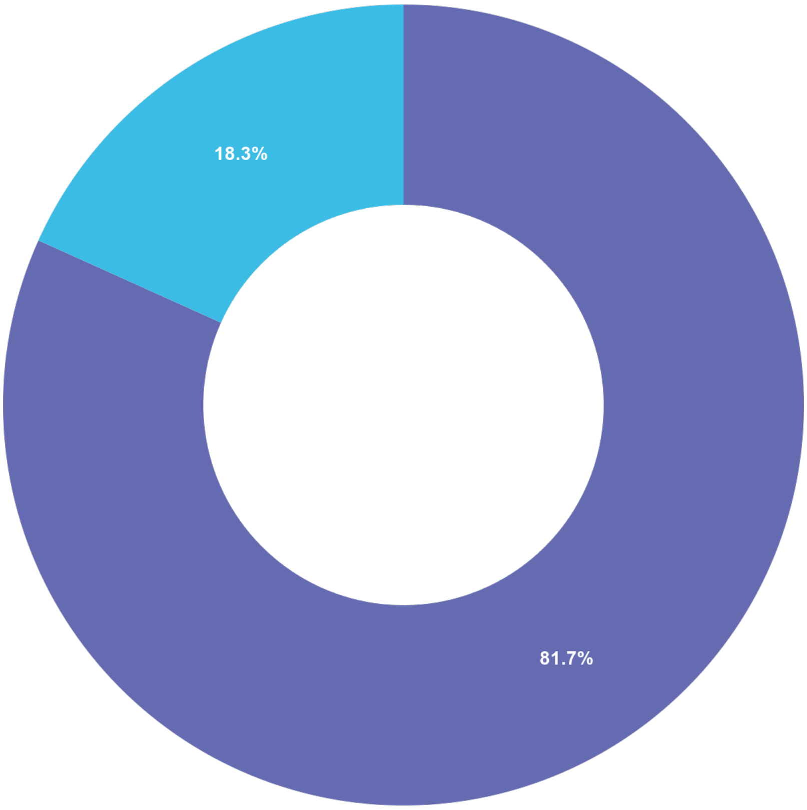

Answered: 115 **Unanswered:** 7

	Choice	Total
a)	Pre-specify clear rules in the protocol	94
b)	On an ad hoc basis	21

Q15 Q15/19: If strata are removed or pooled on an ad-hoc basis just for interim analysis, do you consider this to be the same estimand as the pre-specified one for

the final analysis?

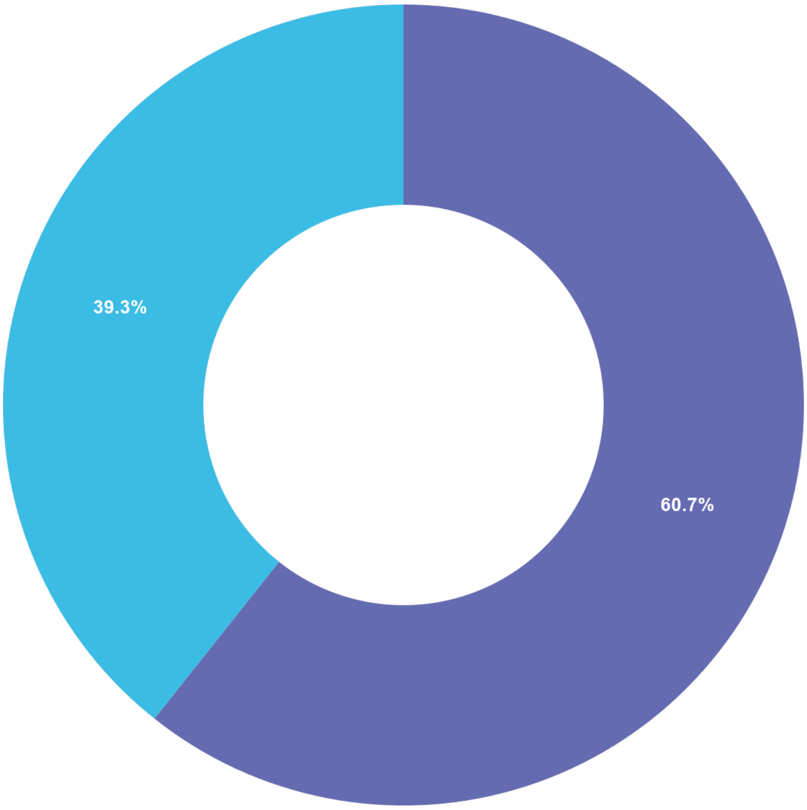

Answered: 117 **Unanswered:** 5

	Choice	Total
	a) Yes	71
	b) No	46

Q16 Q16/19: Have you received generally consistent feedback from multiple regulatory agencies regarding covariate adjustment or stratified analysis? Select all that apply.

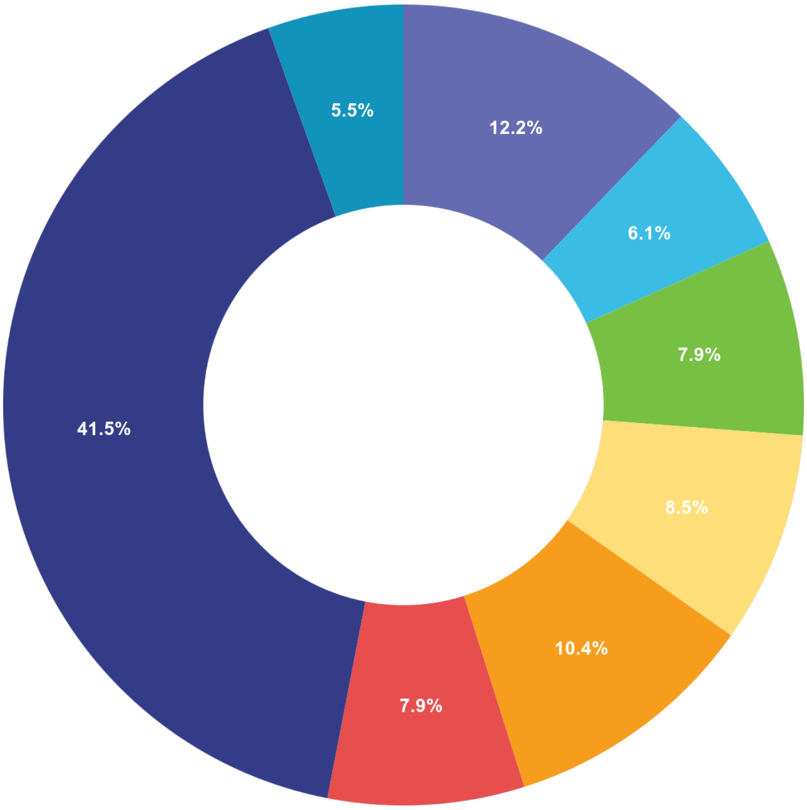

Answered: 108 **Unanswered:** 14

	Choice	Total
	a) Too many strata	20
	b) Small strata	10
	c) Combining small strata	13
	d) Removing stratification factors (that were used in the stratified randomization)	14
	e) Too many covariates in the covariate adjusted analysis	17
	f) The form of covariate variables (e.g. dichotomized from a continuous variable)	13
	g) No	68
	h) Other (please specify the feedback in the comment box)	9

Q17 Q17/19: If you received feedback from regulatory agencies, please provide

more details on what regulatory agency you have engaged (FDA/EMA/PMDA/ NMPA). Select all that apply.

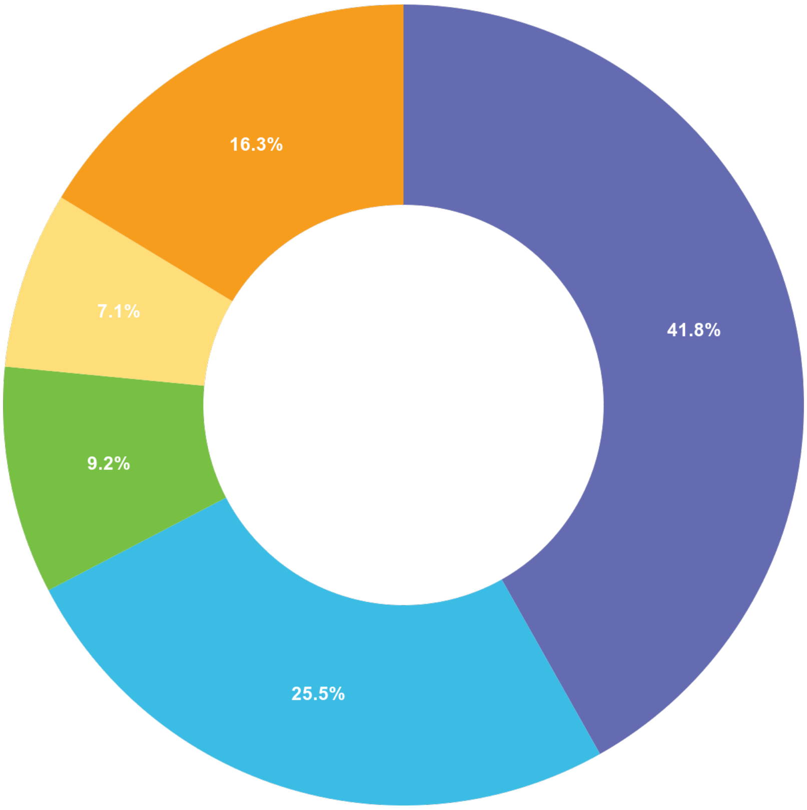

Answered: 60 **Unanswered:** 62

Choice	Total
a) FDA	41
b) EMA	25
c) PMDA	9
d) NMPA	7
e) Other (please specify the regulatory agencies you received feedback in the comment box)	16

Q18 Q18/19: In your experience, what are the most common challenges you have faced when implementing covariate adjustment or stratified analysis in clinical trials? Select all that apply.

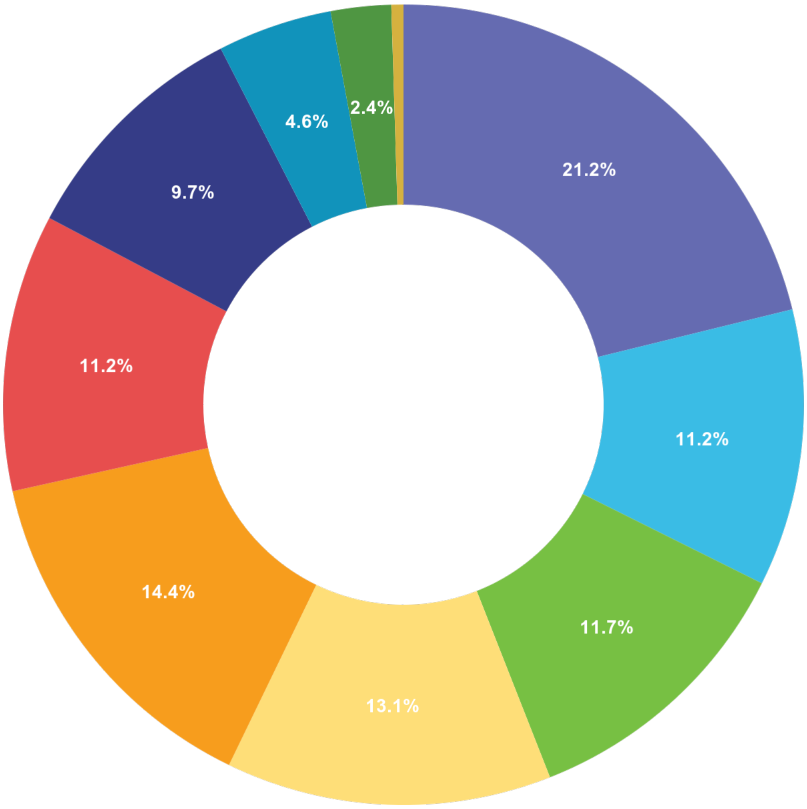

Answered: 114 **Unanswered:** 8

Choice	Total
a) Identifying appropriate covariates or stratification factors	87
b) Determining the optimal number of strata	46
c) Addressing imbalance in strata	48
d) Different results identified among covariate adjustment, stratified analysis and unstratified analysis	54
e) Interpretation of results with complex models	59
f) Communicating results to non-statistical stakeholders	46

	Choice	Total
	g) Type I error and power evaluation, sample size calcuation, especially for covariate adjustment analysis	40
	h) P-value calculation for covariate adjustment	19
	i) Other (please specify your challenges in the comment box)	10
	__archived__	2

Q19 Q19/19 : What resources or support would be most helpful for you in addressing the challenges of covariate adjustment and stratified analysis in clinical trials? Select all that apply.

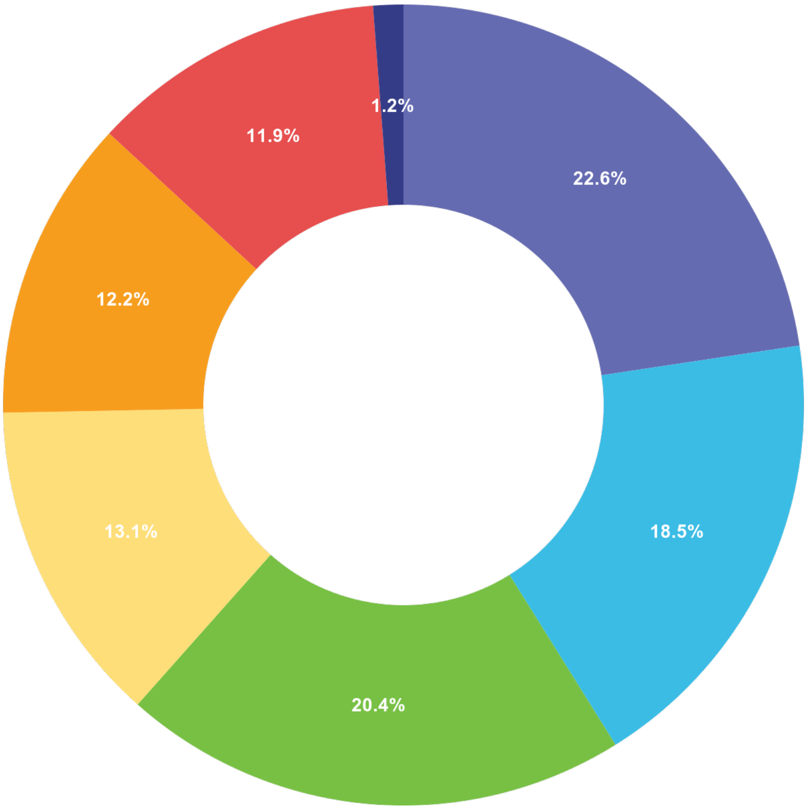

Answered: 116 **Unanswered:** 6

	Choice	Total
	a) Access to comprehensive guidelines or best practices	93
	b) Webinars or training sessions on specific topics	76
	c) Case studies or examples from industry experts	84
	d) A platform for collaboration and discussion with fellow statisticians	54
	e) Consultation or mentorship from experienced professionals	50
	f) Access to specialized software or tools for covariate adjustment and stratified	49
	g) Other (please specify your needs in the comment box)	5
